# Functional Materials and Innovative Strategies for Wearable Thermal Management Applications

**DOI:** 10.1007/s40820-023-01126-1

**Published:** 2023-06-29

**Authors:** Yeongju Jung, Minwoo Kim, Taegyeom Kim, Jiyong Ahn, Jinwoo Lee, Seung Hwan Ko

**Affiliations:** 1 Applied Nano and Thermal Science Lab, Department of Mechanical Engineering, Seoul National University, 1 Gwanak-ro, Gwanak-gu, Seoul, 08826 South Korea; 2 Department of Mechanical, Robotics, and Energy Engineering, Dongguk University, 30 Pildong-ro 1-gil, Jung-gu, Seoul, 04620 South Korea; 3 Institute of Advanced Machinery and Design (SNU-IAMD), Seoul National University, Gwanak-ro, Gwanak-gu, Seoul, 08826 South Korea; 4 Institute of Engineering Research, Seoul National University, 1 Gwanak-ro, Gwanak-gu, Seoul, 08826 South Korea

**Keywords:** Thermal management, Passive heat transfer, Active heat transfer, Wearable materials, Wearable device

## Abstract

**Highlights:**

- This article systematically reviews the thermal management wearables with a specific emphasis on materials and strategies to regulate the human body temperature.
- Thermal management wearables are subdivided into the active and passive thermal managing methods.
- The strength and weakness of each thermal regulatory wearables are discussed in details from the view point of practical usage in real-life.

**Abstract:**

Thermal management is essential in our body as it affects various bodily functions, ranging from thermal discomfort to serious organ failures, as an example of the worst-case scenario. There have been extensive studies about wearable materials and devices that augment thermoregulatory functionalities in our body, employing diverse materials and systematic approaches to attaining thermal homeostasis. This paper reviews the recent progress of functional materials and devices that contribute to thermoregulatory wearables, particularly emphasizing the strategic methodology to regulate body temperature. There exist several methods to promote personal thermal management in a wearable form. For instance, we can impede heat transfer using a thermally insulating material with extremely low thermal conductivity or directly cool and heat the skin surface. Thus, we classify many studies into two branches, passive and active thermal management modes, which are further subdivided into specific strategies. Apart from discussing the strategies and their mechanisms, we also identify the weaknesses of each strategy and scrutinize its potential direction that studies should follow to make substantial contributions to future thermal regulatory wearable industries.
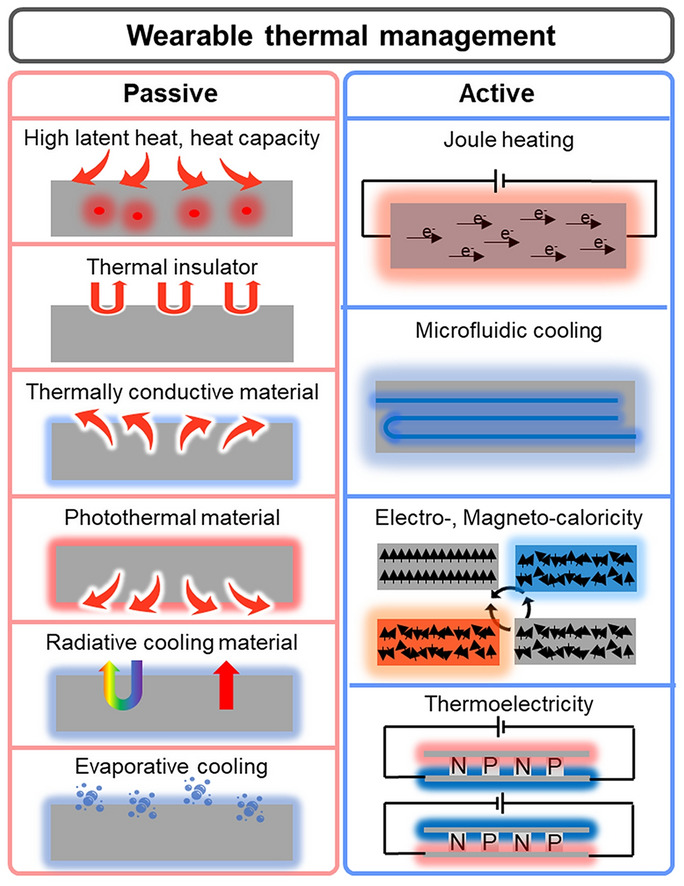

## Introduction

The recent advance in nanomaterials and manufacturing processes facilitated the emergence of smart wearables and many wearable applications. Wearable thermal management has received considerable attention in various academic and industry fields because thermal management is deeply associated with homeostasis in our body. Our bodies generate heat, and we rely on our environment to help us dissipate that heat through sweating and radiation to maintain homeostasis. Still, it becomes problematic if the environment temperature deviates largely from our body temperature. For instance, severe dysfunction of thermoregulation in our body harms enzymatic activities and can even lead to multiple organ failures in extreme cases [1, 2]. Besides serious thermoregulatory malfunctioning of the body, thermal discomfort affects our performance and productivity in everyday life. In this regard, air conditioners and heat pumps have been used to regulate the indoor temperature of buildings and vehicles. Still, air conditioners and heat pumps utilize refrigerants such as chlorofluorocarbons (CFCs) and hydrochlorofluorocarbons (HCFCs) that adversely impact the environment [3].

Thus, many alternatives have been developed to promote human thermal management in a wearable form due to the recent advancements in material science and wearable technologies. Instead of regulating the indoor atmosphere temperature, regulating the body temperature directly is rather effective. Therefore, researchers devised various innovative thermal management methods to control body temperature, including nanomaterials to devices with novel functionalities. There are a considerable number of review articles on thermal management wearables. Still, no review paper has systematically categorized thermal management into methodologies. However, several strategies exist to control the body temperature besides simply cooling and heating the human body.

This paper reviews the recent advances in wearable thermal management materials and innovative strategies to help regulate human body temperature. To present them systemically, we categorized thermoregulatory wearables into active and passive thermal management, further subdivided into various strategies of functional materials and devices. The paper discusses the strengths and limitations of each material/device that constitutes each strategy, and then it concludes with the future perspective and challenges of thermal regulatory wearable technologies.

## Strategies for Wearable Thermal Management

**Fig. 1:**
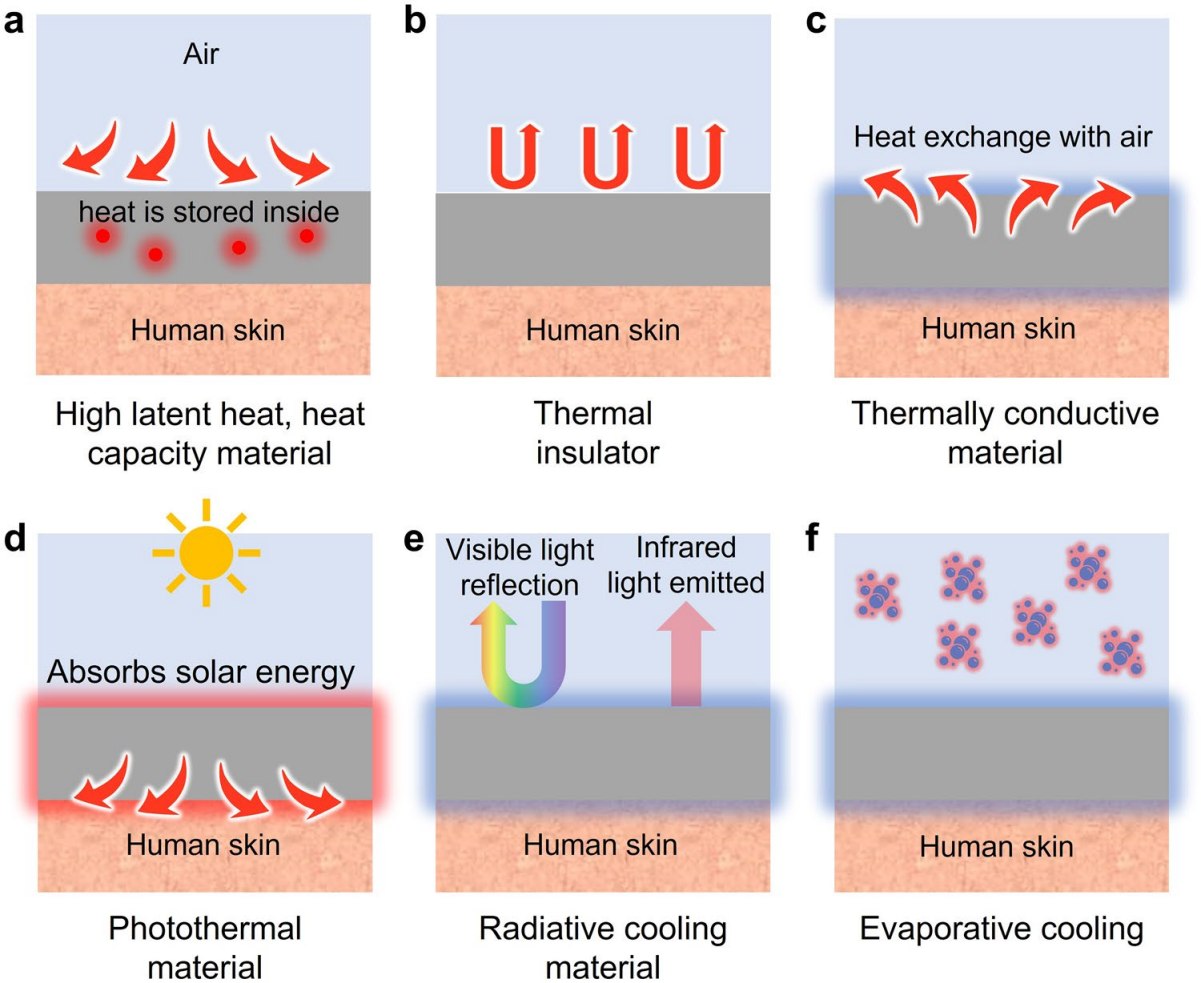
Passive thermal management methods: **a** high latent heat or high heat capacity materials to store heat from the external environment. **b** Thermal insulator that minimizes heat transfer to the human skin. **c** Thermally conductive material that exchanges heat with the air. **d** Photothermal material that absorbs solar energy and heats up the human skin. **e** Radiative cooling material that refrigerates the human skin by reflecting visible light and emitting infrared light toward space. **f** Evaporative cooling materials that facilitate liquid-to-vapor transition

Figure 1 describes various strategies to artificially regulate the human body temperature in passive modes. Figure 1a presents the first strategy of passive thermal management: materials with high latent heat and heat capacity to absorb/release the heat from/to the surroundings such that the external heat is not transferred to the human body. These include a variety of polymers and phase-change materials (PCMs) that absorb/release a substantial amount of heat at a certain temperature. Since PCMs can absorb a vast amount of heat, utilizing PCMs as thermal management materials can be desirable. However, PCMs have a specific phase change temperature, which limits their effectiveness in a wide range of temperature because PCMs would not be able to absorb/release energy if the ambient temperature does not reach the phase change temperature. Another method of passive thermal management is to exchange heat with the surrounding environment by employing materials with high thermal conductivity, including metals and conductive polymer nanomaterials that possess exceptional thermal conductivity (Fig. 1b). Using thermally conductive materials to facilitate heat transfer from/to the surrounding environment can be an effective strategy. Nevertheless, since they follow a passive mechanism, they might transfer heat to/from the human body even when thermal management is not desired. Conversely, inhibiting the heat transfer from the external environment to the human body can be an alternative strategy to achieve thermal management, as in Fig. 1c. It includes a variety of thermal insulators and innovative nanostructures that hinder heat transfer. Although insulating heat transfer can offer a potent solution to attain thermal management, the thermal insulators might cause problematic issues since they can reduce breathability and trap excess heat, which can rather cause discomfort. Furthermore, photothermal materials can heat up by themselves during the daytime in sunlight. These materials collect light energy and convert it into thermal energy, as shown in Fig. 1d, providing thermal management without any electrical power. Nonetheless, the use of the photothermal effect might involve a serious disadvantage since it can elevate the body temperature and even cause a skin burn as the photothermal effect occurs constantly. On the other hand, radiative cooling does the exact opposite of photothermal materials. It can cool down the human body temperature by reflecting visible light while emitting mid-infrared light to space (Fig. 1e). Radiative cooling does not require electricity to generate cooling since it transfers heat to the space by radiation. Yet, just as the photothermal effect, it might cause thermal discomfort because radiative cooling occurs constantly and cannot be controlled. Lastly, evaporative cooling can regulate body temperature as a passive thermal management strategy. These materials artificially facilitate sweat evaporation and decrease the body temperature since sweat evaporation takes energy with it in heat. Specifically, the heat energy from the skin is used to convert the liquid sweat into water vapor. Although evaporative cooling offers an effective tool to regulate body temperature, evaporative cooling suffers from several limitations such as limited effectiveness in high humidity conditions and increased water loss in the human body.

**Fig. 2:**
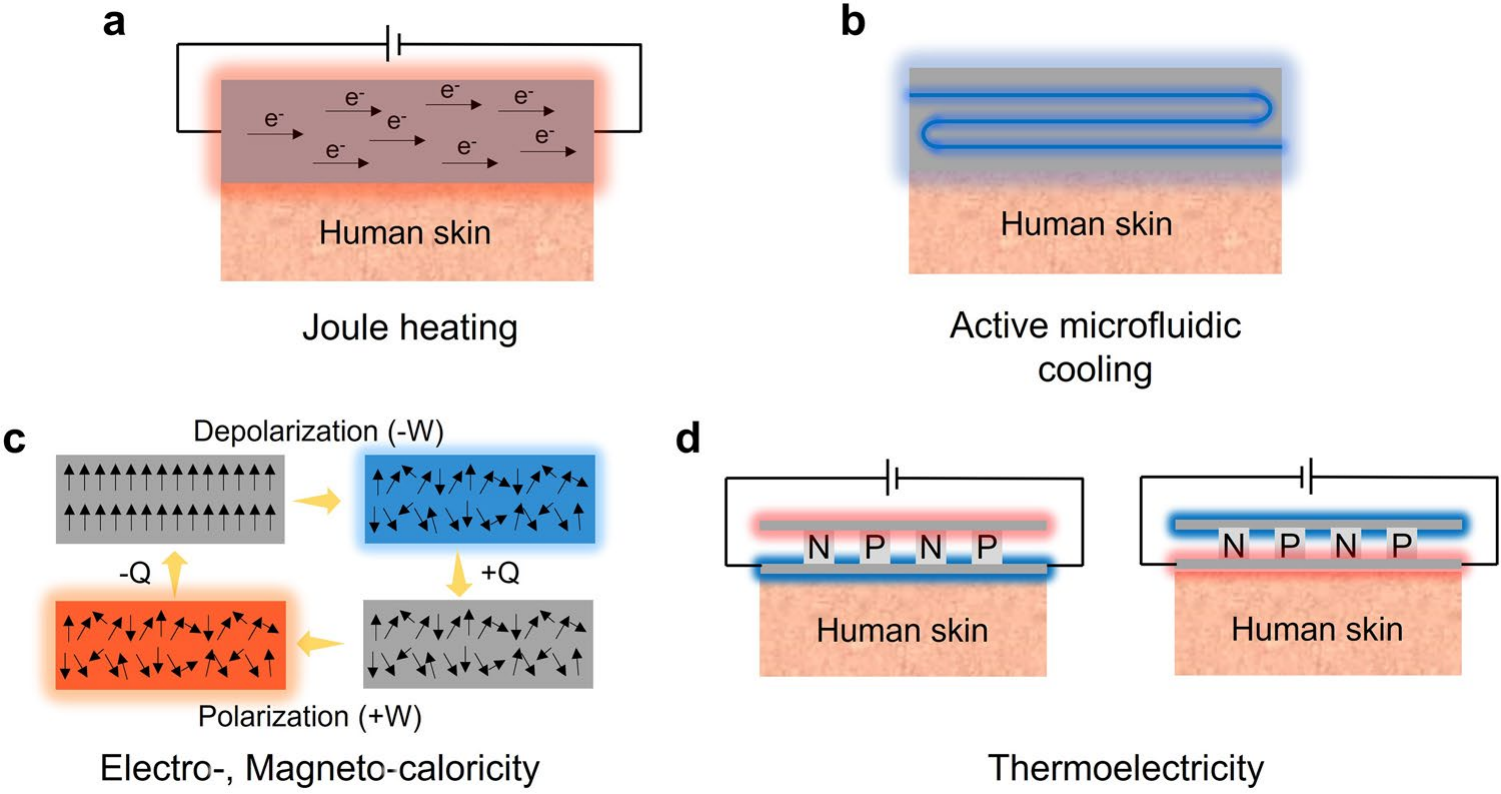
Active thermal management methods: **a** Joule heating. **b** Active microfluidic cooling. **c** Electro-, magnetocaloric cooling and heating. **d** Thermoelectric device cooling and heating

Figure 2 mainly addresses wearable electronics that can cool or heat by actively transferring heat from/to the human body with external input. Figure 2a delineates the mechanism of Joule heating that serves to actively heat in the presence of electrical input. Joule heating is based on the principle that the flow of electrons experiences resistance when they encounter obstacles in the conductor's atomic structure, such as impurities or defects. As the electrons encounter resistance, they lose some of their kinetic energy, which is converted into heat energy. Due to the simple working mechanism, there have been a great number of works on wearable Joule heaters, and Joule heaters can be fabricated in a highly stretchable and thin form such that they can make intimate contact with the skin surface for efficient heat transfer. However, the Joule heater can only provide heating and requires an external power source to supply electrical voltage to the device. Microfluidic cooling exemplifies another example of active wearable thermal management, as shown in Fig. 2b. Microfluidic cooling is a mechanism that involves the use of microscale fluid flow to transfer heat away through convective heat transfer. The development of elastomeric polymer and microfluidic channel manufacturing techniques enables the application of microfluidic cooling for wearable purposes. Depending on the fluid temperature, the microfluidic channels can cool or even heat the human body, but just as Joule heating, they require the external pump to facilitate the fluid circulation, and the incorporation of the pump might aggravate the wearability of the device. Figure 2c illustrates the mechanism of electrocaloric and magnetocaloric devices that induce a reversible temperature change in a material using the electric (electrocaloric) or magnetic (magnetocaloric) fields, respectively. In the process, the entropy of material changes in response to an applied electric/magnetic field, leading to a temperature change, which can be used to cool or heat an arbitrary object, depending on the direction of the applied field. However, one of the most critical limitations of electrocaloric and magnetocaloric devices for wearable applications arises because it requires an additional actuating system to constantly transfer heat in one direction to cool or heat actively. Such a drawback of electrocaloricity and magnetocaloricity limits its widespread use in wearable thermal management applications. We will further discuss its limitations and exemplary studies that overcame such weaknesses in the following section. Lastly, Fig. 2d illustrates the mechanism of thermoelectric devices, which utilizes electrical input to generate active cooling and heating, as electrocaloric and magnetocaloric devices do. However, unlike these devices, thermoelectric devices follow a completely different physical mechanism to induce the temperature difference. In the presence of the electrical voltage, the charged carriers, such as holes and electrons, instantly diffuse to one side of the device, so the device side, which is densely populated with holes and electrons, starts to heat up due to the vibrational energy of the charged carriers, and the other side cools down. Also, unlike many devices and materials that can only cool or heat with a single device structure, the thermoelectrical device can both cool and heat an arbitrary object or space simply by reversing the direction of the electrical current. For this reason, there exists a great number of stretchable and flexible thermoelectric devices that are developed to actively regulate the human body temperature, but just as other active thermal management methods, they require an external power source for electricity input and suffer from low cooling efficiency [4]. Tables 1 and 2 summarize representative works on each mechanism of passive and active thermal management wearables.

**Table 1:** Passive thermal management wearables

References	Active/ passive	Heat transfer mechanism	Capability to cooling and heating	Thermal management performance	Energy consumption	Flexible/ stretchable	Breathability
[5]	Passive	Latent heat storage	Heating	High thermal conductivity (5.34 W m^−1^ K^−1^), high enthalpy (125.2 J g^−1^)	No	Flexible	
[6]	Passive	Latent heat storage	Heating	N/A	No	Flexible/ stretchable	No
[7]	Passive	Latent heat storage	Cooling & heating	Phase change temperature from 5 to 60 °C with varying PEG molecular weights and high latent heat (118.7 J g^−1^)	No (Yes for electrical heating)	Flexible	No
[8]	Passive	Latent heat storage	Cooling & heating	High dimension retention ratio (98.1%) and latent heat value (163.3 J g^−1^)	No	Flexible/ stretchable	Breathable
[9]	Passive	Latent heat storage	Cooling & heating	Latent heat (158.65 J g^−1^) and economic benefits (4.85 × 10^−3^ ¥ J^−1^)	No	Flexible	No
[10]	Passive	Heat conduction	N/A	Thermal conductivity of 1.37 W m^−1^ K^−1^	No (Yes for sensing)	Flexible/ stretchable	Breathable
[11]	Passive	Heat conduction	N/A	Thermal conductivity (20–30 W m^−1^ K^−1^)	No	Flexible	No
[12]	Passive	Latent heat storage & heat conduction	Cooling & heating	Enthalpy of 206.0 J g^−1^	No	Flexible	No
[13]	Passive	Latent heat storage & heat conduction	N/A	Thermal diffusivity of 0.307 mm^2^ s^−1^ and latent heat of 94.29 J cm^−3^	No	Stretchable	No
[14]	Passive	Thermal insulation	N/A	Low thermal conductivity of 0.031 W·m^–1^ K^–1^ and high heat resistance (> 500 °C)	No	Stretchable	No
[15]	Passive	Thermal insulation	N/A	N/A	No	Flexible	No
[16]	Passive	Thermal insulation	N/A	Temperature-invariant compression resilience from − 196 to 1000 °C, and thermal conductivity as low as 0.034 W·m^−1^ K^−1^	No	Stretchable	Breathable
[17]	Passive	Thermal insulation	N/A	High-temperature resistance < 1,300 °C and low thermal conductivity of 0.0322 W m^−1^ K^−1^	No	Flexible	No -
[18]	Passive	Thermal insulation	N/A	Temperature-invariant superelasticity from − 196 to 1100 °C, low thermal conductivity of 0.0223 W m^−1^ K^−1^	No	Flexible	No -
[19]	Passive	Thermal insulation	N/A	Excellent thermal stability at temperatures as high as 1200 °C in butane blow torch or as low as − 196 °C in liquid nitrogen and a thermal conductivity of 28.4 mW m^−1^ K^−1^	No	Stretchable	No -
[20]	Passive	Photothermal effect	Heating	Temperature increase of ∼111 ± 2.6 °C after the application of 600 mW cm^–2^ light irradiation for 5 min a high optical transmittance of ∼83%	No	Stretchable	No
[21]	Passive	Photothermal effect	Heating	Temperature increases of 60 °C	No	Flexible	No
[22]	Passive	Photothermal effect	Heating	19.7 °C increase with a light intensity of 1,000 W m^−2,^	No	Stretchable	No
[23]	Passive	Photothermal effect	Heating	Light absorbance of > 95% from ultraviolet to far infrared range	No	Flexible	No
[24]	Passive	Photothermal effect	Heating	Equilibrium temperature of 65.4 °C under one-sun illumination	No (Yes for electrical heating)	Stretchable	No
[25]	Passive	Photothermal effect	Heating	Passive radiative heating (4.9 °C higher than conventional cotton), Solar heating (73.5 °C)	No (Yes for electrical heating)	Flexible	Breathable
[26]	Passive	Sweat evaporation & heat conduction	Cooling	50% higher evaporation rate (1.6 mL h^−1^) than conventional fabrics	No	Flexible	Breathable
[27]	Passive	Sweat evaporation	Cooling	Sweating rate (520 mL h m^2^ h^−1^) ~ 32.3 °C (on the other hand, the temperature of skin covered with normal wicking layer ~ 35.9 °C)	No	Flexible	Breathable
[28]	Passive	Sweat evaporation	Cooling	Forward transportation capability of 1,115%, Backward transportation capability of − 1509%	No	Flexible	Breathable
[29]	Passive	Sweat evaporation & heat conduction	Cooling	one-way transport index (1072%), water evaporation rate (0.36 g h^−1^)	No	Stretchable	Breathable
[30]	Passive	Sweat evaporation and heat conduction	Cooling	3 times higher skin power density (d*q*/d*v*) increment than conventional cotton, ~ 3 °C lower than the human body covered with cotton	No	Flexible	Breathable
[31]	Passive	Radiative cooling	Cooling	~ 8, ~ 12.5, ~ 19 °C lower than the same skin covered with natural silk or cotton or left uncovered, respectively	No	Flexible	Breathable
[32]	Passive	Radiative cooling	Cooling	~ 4.8 °C lower than the human body covered with commercial cotton fabric	No	Stretchable	Breathable
[33]	Passive	Radiative cooling & photothermal	Cooling & Heating	Cooling: 3.7 °C lower than the skin simulator covered with white cotton, Heating: 6.2 °C higher than the skin simulator covered with black cotton	No	Flexible	Breathable
[34]	Passive	Radiative cooling & sweat evaporation	Cooling	~ 4.2 °C lower than the human body covered with commercial cotton textile	No	Flexible	Breathable
[35]	Passive	Radiative cooling & sweat evaporation	Cooling	~ 16.6 °C lower than the commercial textiles, including a contribution from sweat management (~ 8.2 °C)	No	Flexible	Breathable
[36]	Passive	Radiative cooling & sweat evaporation	Cooling	~ 2.6 °C lower than that of cotton without perspiration ~ 1.0 °C lower than that of cotton only with evaporation cooling	No	Flexible	Breathable
[37]	Passive	Radiative cooling & Sweat evaporation	Cooling	~ 21.9 °C lower than the traditional cotton-covered skin simulator	No	Flexible	Breathable

**Table 2:** Active thermal management wearables

References	Active/ passive	Heat transfer mechanism	Capability to cooling and heating	Thermal management performance	Energy consumption	Flexible/ stretchable	Breathability
[38]	Active	Heat conduction	Heating	272 °C at 2.5 V, 89 °C at 1.4 V where environmental temperature was − 30 °C, saturation time of 6 s	0.85–4.25 W	Stretchable	No
[39]	Active	Heat conduction	Heating	Up to 80 °C depending on input voltage	0.11–4.05 W	Stretchable	Yes
[40]	Active	Heat conduction	Heating	Up to 45 °C by safety feedback control@@100 °C at 6 V, saturation time 16 s	1.0–1.6 W	Flexible	Yes
[41]	Active	Heat conduction	Heating	Up to 40 °C by considering conduction, saturation time 1–2 s, 120 °C at 7 V	1.0–6.0 V	Stretchable	Yes
[42]	Active	Heat conduction	Heating	saturation time < 0.6 s by active control, target temperature 25.5 to 36 °C	1.94 W for blue color	Flexible	No
[43]	Active	Heat conduction/ convection	Both	3–58 °C for robot hand@@27 s to phase transit solid gallium microgranule to liquid@@49 s from liquid to solid	N/A (liquid injection)	Flexible	No
[44]	Active	Heat conduction/ convection	Cooling	− 1.4 °C for nerve temp, maximum cooling rate of 3 °C s^−1^	N/A (liquid injection)	Flexible	No
[45]	Active	Heat conduction/ convection	Cooling	35.3 to 20.3 °C, saturation time 300 s	N/A (liquid injection)	Flexible	No
[46]	Active	Heat conduction/ convection	Both	N/A	16 μW cm^-2^ (generation)	Flexible	No
[47]	Active	Heat conduction	Both	ΔT = 10.1 K, Q = 5.0 kJ kg^−1^ at room temp (under 60 MV m^−1^)	N/A	Flexible	No
[48]	Active	Heat conduction	Both	ΔT = 22.5 K at room temp (under 1,200 kV cm^−1^)	N/A	Flexible	No
[49]	Active	Heat conduction	Both	ΔT = 12 K at room temp ΔT/ΔE = 0.007 K cm kV-1	N/A	Flexible	No
[50]	Active	Heat conduction	Both	ΔT = 13.99 K at -30 °C (under 1500 kV cm^−1^), ΔT = 5.08 K at 70 °C (under 1500 kV cm^−1^),	N/A	Flexible	No
[51]	Active	Heat conduction	Both	ΔT = 6 K (under 600 kV cm^−1^) with high thermal conductivity, saturation time 13.0 to 17.8 s	N/A	Flexible	No
[52]	Active	Heat conduction	Both	ΔT = 15 K, saturation time 3.52 s for heating, 4.48 s for cooling with active control	0.2–1.5 A	Stretchable	No
[53]	Active	Heat conduction	Both	10–50 °C for cloaking saturation time 5.1 s for heating, 6.4 s for cooling with active control	0.6–1.5 A	Flexible	No
[54]	Active	Heat conduction	Both	N/A	7.02 mW (generation)	Stretchable	No
[55]	Active	Heat conduction	Both	≈ 34 °C by PID controlled thermoregulation	0.7—2 A	Stretchable	No

## Passive Thermal Management

### High Latent Heat Storage

Latent heat storage (LHS) stores heat in a storage medium in the form of potential energy between the particles of the substance. Converting heat to potential energy within a substance involves a phase change, resulting in heat storage without significant changes in the storage medium's temperature. The capacity of latent heat storage can be calculated using the following method:

1 $$Q=m\gamma $$

where *γ* is latent heat in kJ/kg and *m* is the mass in kg. The thermal storage density of the storage medium can be expressed below:

2 $$\frac{Q}{V}=\rho \gamma $$

where $V$ and $\rho$ are volume and density in cm^3^ and kg cm^−3^, respectively.

PCMs are increasingly used for latent heat storage (LHS) due to their ability to store a large amount of heat during phase transition, providing superior thermal stability and compatibility. LHS is a high-energy density storage technology that can maintain a constant temperature that matches the phase transition temperature of the PCM. This confers a significant advantage over other thermal energy storage technologies, as it can store more heat and better stabilize the temperature [56, 57]. LHS has a wide range of applications in various fields, including heating and cooling systems [58–63], where it can store thermal energy during low-demand hours and release it when needed, thus reducing energy consumption and costs during high-demand hours. Another promising application is in solar thermal energy storage [64–66], where LHS can store solar energy collected during the daytime and release it at night or during cloudy days, providing a reliable energy source for heating and electricity generation.

**Fig. 3:**
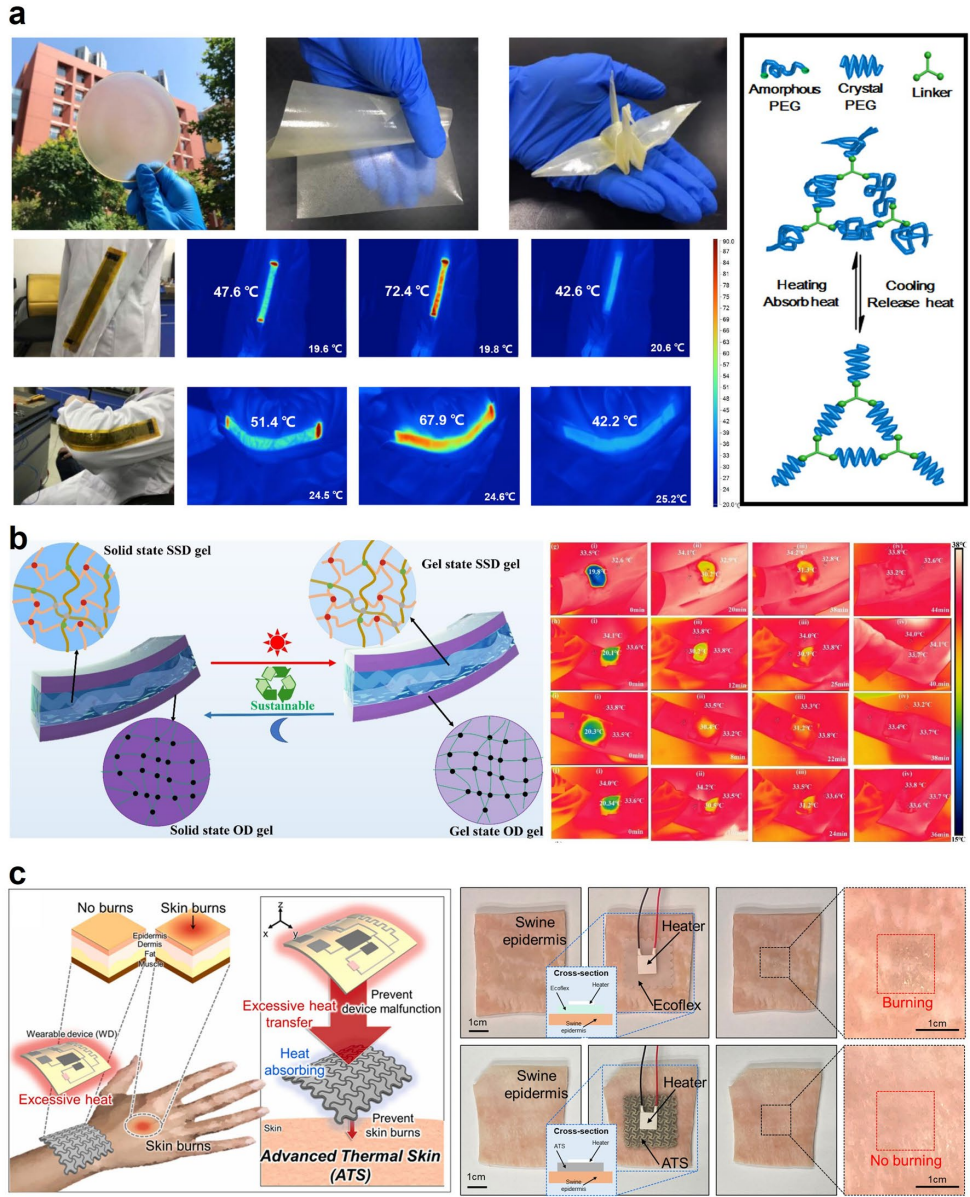
Heat storage-based personal thermal management: **a** Phase-change hydrogels with thermal energy storage for personal healthcare. Reproduced with permission [7]. Copyright 2022, Elsevier. **b** Self-healable thermal energy storage for personal thermal management. Reproduced with permission [9]. Copyright 2023, Elsevier. **c** High heat storage and thermal diffusivity-based thermoregulation. Reproduced with permission [13]. Copyright 2023, Elsevier

Recently, latent heat storage (LHS) technology has been explored for thermal management in wearable devices [5, 6, 67]. However, the use of phase change materials (PCMs) in this application remains challenging due to their solid rigidity and potential for liquid leakage. To address these issues, Qi et al. developed a new method for thermal management in wearable devices using a flexible PCM film to address the issues of solid rigidity and liquid leakages of conventional PCMs, as in Fig. 3a. This involved synthesizing large-area intrinsic PCM films (MTPEG) with excellent self-support, ultra-flexibility, and shape-conformability through chemical tailoring melamine and toluene-2,4-diisocyanate (TDI) with polyethylene glycol (PEG) materials. The integration of flexible PCM films and a flexible graphene film as a thermal source can result in exceptional temperature control and electro-thermal and photothermal energy conversion performance. For example, the PCM film developed in this work shows the controllable phase change temperature between 5 and 60 °C by adjusting the chemical composition of PEG. Also, the PCM herein exhibited a high latent heat of 118.7 J g^−1^ and remained intact even after 1,000 heating cycles of the solid–solid transitions. To demonstrate the high energy-storing capability of the proposed PCM, the authors integrated the PCM into the flexible graphene film and applied the electrical current of 1.5 A. The result showed high electro-thermal energy conversion efficiency of 94% and substantiated its potential usage in wearable thermal management applications [7].

Another work on the flexible PCM by Luo et al. presents the sandwich-structured thermal energy storage composite based on two different PCM gel materials, as shown in Fig. 3b. The authors encapsulated the inorganic PCM (sodium sulfate decahydrate) with the organic PCM (octadecane) to attain high-form stability, cyclic stability, self-repairing, biocompatibility, and latent heat. The encapsulating design that confines sodium sulfate decahydrate endows the material composite with biocompatibility since sodium sulfate is known to be toxic due to high concentrations of salts. As a heat-storing material, the resultant composite exhibits a high latent heat of 158.65 J g^−1^ without supercooling. Apart from the thermal characteristics of the PCM, the material shows high form-stability without leakage of inorganic PCM that arises from the crosslinking of the inorganic PCM and physical encapsulation of the organic PCM. Also, the material did not show a substantial degradation in thermal performance even after 200 cycles of heating and cooling. Moreover, the material developed in this work exhibits the self-repairing feature based on chemical bonds such as hydrogen, ionic, and Van der Waals bonds that contribute to physical reattachment to the corresponding counterpart. To corroborate its potential applications to thermoregulatory wearables, the authors validated the heat-storing capability of PCM by mounting the PCM composite and examining the body temperature [9].

Besides the outstanding works on the heat-storing capabilities of PCMs, our group recently demonstrated the human skin-like material composite that exhibits both high thermal diffusivity and heat storage capability, as shown in Fig. 3c. Despite the favorable thermal properties of phase change materials (PCMs) for wearable thermal management, their low thermal diffusivity is a significant intrinsic limitation. This low thermal diffusivity hinders heat diffusion within the PCM matrices, leading to the concentration of external heat at localized spots without dispersion to the rest of the PCM matrices. To address this issue of the conventional PCMs, the study presented a novel interfacial layer between the skin and wearable device, which consists of a serpentine structure made of silver flake and polydimethylsiloxane (SPS) and a sodium-acetate-based hydrogel matrix (SAHM). The high thermal diffusivity of SPS ensures an even distribution of heat, while the high thermal storage capability of SAHM enables heat absorption without a significant temperature increase. This layer offers both thermal protection to the skin and prevention of device malfunctioning by efficiently absorbing the heat released from the device. Also, owing to the incorporation of hydrogel matrix into the composite, SAHM is found to retain a small modulus change of 4.8 fold while maintaining comparable heat capacity to paraffin (94.29 J cm^−3^), distinguishing it from typical PCMs and thus addressing the intrinsic limitation of the high modulus change between the liquid and solid phases. The phase-independent softness of the PCM in this work promotes conformal contact with deformable surfaces and enhances the potential for wearable thermal management applications [13]. As discussed, the heat storage materials, especially PCMs, exhibit the desirable features to be applied to the heat regulatory wearables as they can absorb or release heat depending on the temperature gradient. Some recent works, as mentioned above, addressed the intrinsic limitations of conventional PCMs, such as mechanical modulus variation between phases, leakage, and low thermal diffusivity. Nevertheless, further research needs to be done on enhancing the latent heat because the temperature of PCM starts to change after the phase transition. It can cause an undesirable heat transfer between the human body and the external environment.

### Thermally Conductive Material

Thermally conductive materials play a crucial role in regulating the temperature of objects by exchanging heat with the surrounding medium, following the equation below:

3 $$q=-k\nabla T$$

where $q$, $k,$ and $\nabla T$ correspond to local heat flux density, thermal conductivity, and temperature gradient (in this case of thermal management application, the temperature gradient between the body temperature and the surrounding medium), respectively. Recent advancements in nanomaterials and manufacturing technology have led to the development of thermally conductive materials that can elastically deform under stress, making them suitable for wearable applications. By controlling the alignment of the thermally conductive materials, the thermal conductivity of these materials can be tailored to enhance heat transfer in the cross-plane or in-plane directions. Although some studies have not specifically controlled the alignment of the materials [68, 69], aligning them in the cross-plane or in-plane direction is far more effective. For example, materials with high cross-plane thermal conductivity can passively cool the human body temperature by transferring heat from the skin to the external environment [54, 70–84]. On the other hand, materials with high in-plane thermal conductivity can effectively spread heat in the lateral direction, which is particularly useful when the skin temperature of a localized region is too high or low [85–95]. Several fabrication methods are available to control the alignment of thermally conductive materials in both the cross-plane and in-plane directions. One widely used technique to align the thermally conductive materials in the cross-plane direction is to incorporate the magnetic materials into the uncured elastomeric base material and then magnetize the materials while thermal curing such that these materials align in the vertical direction. The filler material for the cross-plane alignment can either have the ferromagnetic characteristic along with high thermal conductivity, as AgNi [54], or the ferromagnetic material can be coated on the surface of the thermally conductive materials [75, 96, 97].

**Fig. 4:**
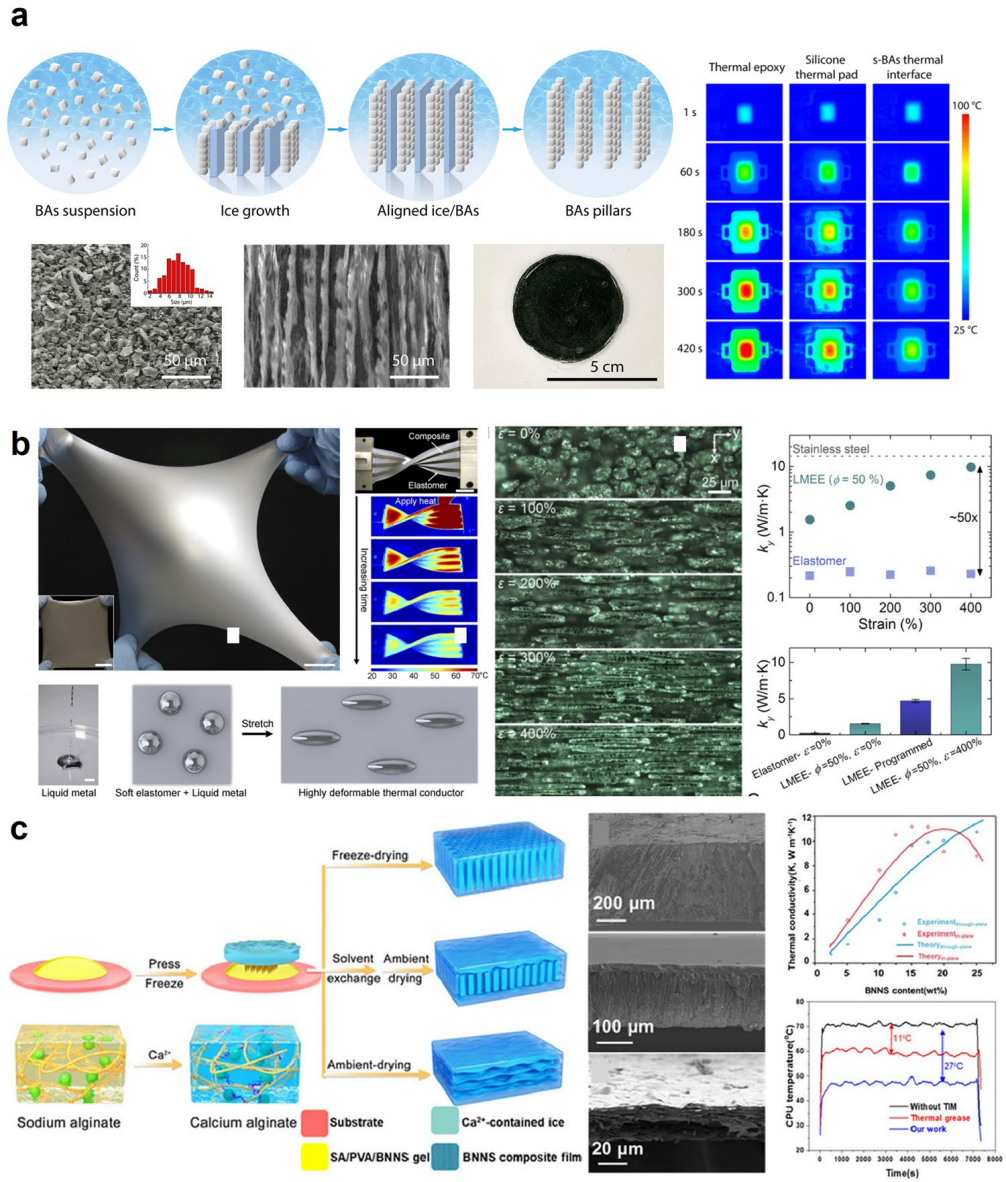
High thermal conductivity materials for passive thermal management: **a** Thermally conductive boron nitride composite that is aligned in the cross-plane direction. Reproduced with permission [98]. Copyright 2021, Springer Nature. **b** Thermally conductive liquid metal-elastomer composite that has the in-plane oriented alignment. Reproduced with permission [21]. Copyright 2017, PNAS. **c** Thermally conductive boron nanosheet composite that can be aligned both in the cross-plane and in-plane direction depending on the manufacturing method. Reproduced with permission [71]. Copyright 2022, Elsevier

Apart from magnetizing the thermally conductive fillers, an interesting study by Cui et al. demonstrated a flexible thermal interface material for high-performance thermal management applications using self-vertically assembled manufacturing of cubic boron arsenides (BAs), as shown in Fig. 4a. Through the rational design of BAs microcrystals in a polymer composite, BAs exhibited highly desirable properties such as high thermal conductivity up to 21 W m^−1^ K^−1^ in the cross-plane direction and excellent elastic compliance similar to soft biological tissues down to 100 kPa. Furthermore, the BAs showed high flexibility and maintained high conductivity over at least 500 bending cycles, opening up new applications for flexible thermal cooling. Furthermore, the group also demonstrated device integration with power LEDs. They measured the superior cooling performance of BAs beyond the current state of the art, with hot spot temperatures reduced by up to 45 °C. This research demonstrates the scalable manufacturing of a new generation of energy-efficient and flexible thermal interfaces, which hold great promise for the advanced thermal management of semiconductors. Although the scope of this research focused on the cooling of electronic devices, the same structure of BA can be directly applied to the human thermal management application since it showed extremely high thermal conductivity in the cross-plane direction with skin-like Young’s modulus [98].

To fabricate the materials with high in-plane thermal conductivity, the researchers resort to specific manufacturing techniques such as vacuum filtration [90], hot-pressing [88], and spraying [85]. An intriguing study investigated the thermal conductivity of a new type of soft elastomers that contain elongated liquid metal inclusions. Incorporating liquid metal into the elastomeric matrix can improve thermal conductivity to a certain extent. Hence, the research group claimed that they could “program” the thermal conductivity of the liquid metal inclusion elastomer by introducing the biaxial strain of 600%. Application of the biaxial strain results in 210% of the plastic deformation of the base elastomer, and such a mechanical deformation boosts up the in-plane thermal conductivity up to 9.8 W m^−1^ K^−1^, which is 50 times greater than the thermal conductivity of the pristine base elastomer. The liquid metal inclusions formed a continuous network throughout the elastomer, allowing for efficient heat transfer by facilitating the movement of heat-carrying phonons. The study suggests that these materials could be useful in a variety of applications where heat dissipation is important, such as in flexible electronics, wearable devices, or soft robotics. The material proposed in this work can also be employed for wearable thermal management applications as it exhibits high thermal conductivity as well as stretchability (Fig. 4b) [21].

A recent study by Huang et al. proposed a manufacturing method that can align the thermally conductive nanomaterials either in both the cross-plane and in-plane directions based on the method called ice-press assembly strategy. The authors created a composite film using boron nitride nanosheets (BNNS) arranged in a ladder-like structure, allowing heat conduction through the BNNSs. To fabricate the film, they used a precursor gel made from an aqueous solution of exfoliated BNNS. It was drop-casted and pressed by frozen ice containing Ca^2+^ to create a temperature gradient. Due to the weight and smooth surface of the ice, the precursor gel was able to spread out as a flat film. Additionally, the low temperature of the frozen ice caused the water in the precursor to solidify in an oriented manner, causing the suspended BNNS and polymer to align along the vertically arranged ice crystal boundaries and form a frozen monolith consisting of oriented ice crystals surrounded by the polymer. Here, Ca^2+^ ions and crosslinking of alginate prevent the composite films from dissolving in ethanol, improving the framework's mechanical properties and preventing collapse during drying. The composite film is frozen and then thawed in ethanol before being immersed in acetone and allowed to dry at room temperature to achieve a ladder-structured BNNS in the composite film. However, without freeze dying, BNNS in a precursor gel is deposited on top of one another, facilitating the heat transfer in the in-plane direction. Thus, the work presented a BNNS composite with anisotropic thermal conductivity generated based on the manufacturing methods. Such a flexible composite obtained in this study exhibits 9.6 and 11.2 W m^−1^ K^−1^ in the in-plane and cross-plane directions [71]. Recent studies also report manufacturing technologies that create alignment of the thermally conductive fillers in both in-plane and cross-plane [99].

Overall, materials with thermal conductivity regulate the body temperature by facilitating heat exchange between the human skin and the external environment. Thus, if the external temperature is lower than the body temperature, heat transfer will occur from the human body to the surrounding environment without external input, which would cool down the body temperature. However, materials with high thermal conductivities will rather exert a negative effect if the surrounding temperature is higher than the body temperature because, in this case, heat will be transferred to the human body from the external environment, so it is advisable to use the thermally conductive materials for the suitable conditions only.

### Thermal Insulation

Thermal insulators are increasingly being considered for various applications, including thermal protection and energy conservation in industries, aerospace, and personal thermal management. Most thermal insulators utilize porous structures to prevent unnecessary heat transfer, because air has lower thermal conductivity than the solid phase, which reduces heat conduction and convection [100]. Aerogel-based materials have been extensively studied for their excellent insulating properties, particularly inorganic aerogels, due to their low thermal conductivity, thermal stability, and high insulating capability. However, challenges remain in using aerogels in a wearable form, mainly their brittleness and fragile properties. Organic aerogels have been studied as a promising alternative due to their outstanding toughness and superior flexibility. Nature-derived materials such as cellulose, chitosan, and polymeric materials have gained significant attention [101]. Nevertheless, the poor stability of organic aerogels at high temperatures limits their potential for widespread applications.

**Fig. 5:**
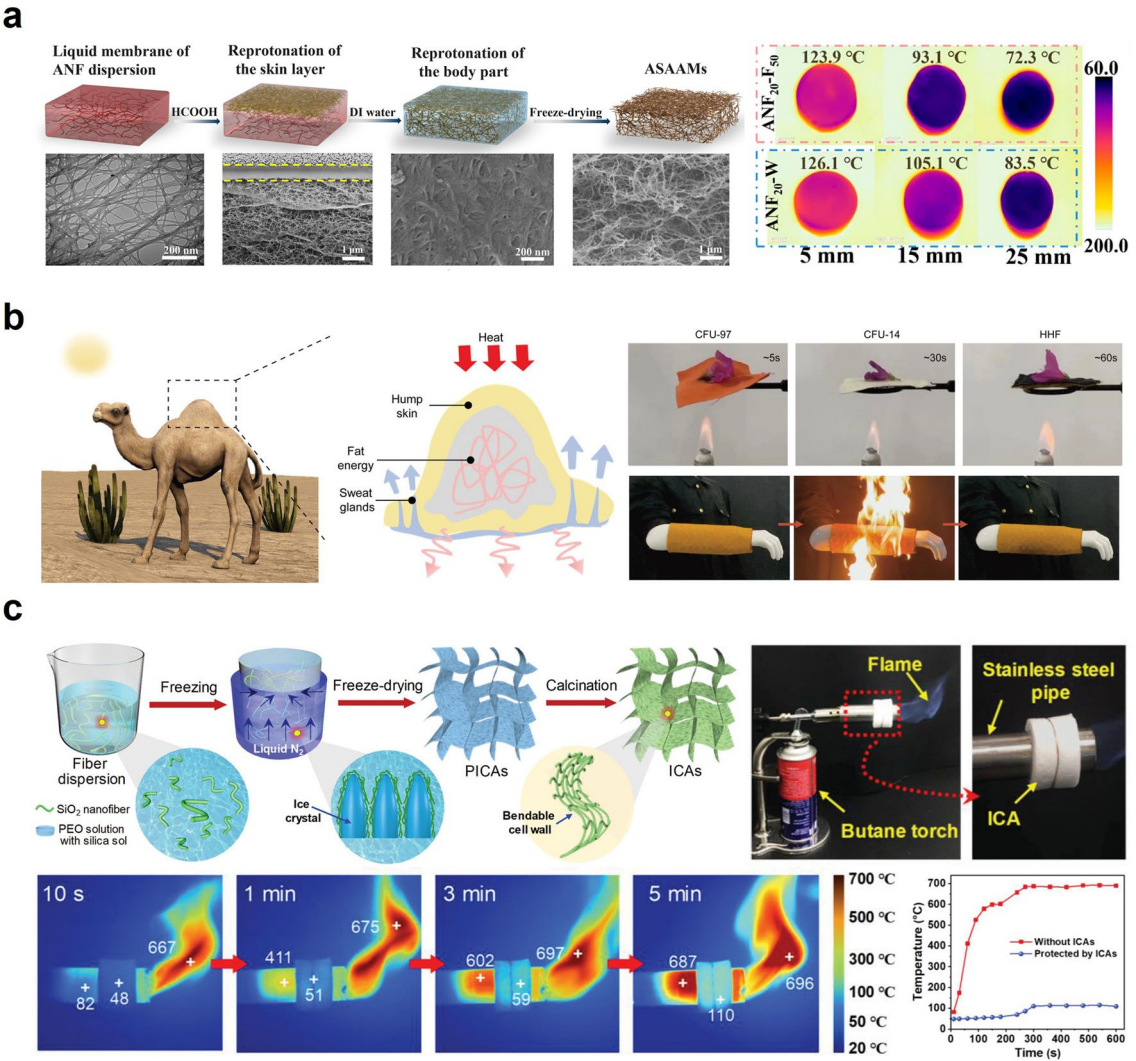
Thermal insulation for passive thermal management: **a** Aramid nanofiber aerogel for high-temperature (> 500 °C) thermal insulation. Reproduced with permission [14]. Copyright 2022, ACS Publications. **b** Hump-inspired fabric for firefighter thermal protection. Reproduced with permission [102]. Copyright 2023, Wiley–VCH. **c** Ceramic nanofiber aerogel with exceptional bendability and compressibility. Reproduced with permission [18]. Copyright 2020, Wiley–VCH

To address the stability issue of organic aerogels at high temperatures, Hu et al. developed aramid nanofiber (ANF) membranes that demonstrate exceptional heat resistance (> 500 °C) [14]. ANFs, typically fabricated from poly(p-phenylene terephthalamide) (PPTA) or Kevlar, have outstanding mechanical properties and excellent thermal stability (Fig. 5a). The researchers created asymmetric porous ANF membranes by subjecting ANFs to protonation processes using formic acid (HCOOH) and DI water as proton donors. The aerogels exhibited an enhanced tensile strength of 11.8 MPa (16.5 times) with an elongation break of 15% and could endure a load of 500 g, which is 3,600 times its weight, owing to their asymmetric structure. In addition, the ANF aerogel demonstrated a low thermal conductivity of 0.0031 W m^−1^ K^−1^, which is lower than that of commonly-used flexible insulators such as polystyrene foam, cellulose aerogel, and mineral wool. The aerogels did not shrink or deform after being heated for 10 s on fire, indicating that the carbonization of the dense layer would further block the burning of the inner ANFs. These excellent properties, such as low thermal conductivity and flame-retardancy, make ANF membranes a potential thermal insulator for harsh environments.

Likewise, most of the previous research literature has concentrated on wearable thermal insulators, which can further be utilized at elevated temperatures, yet still lack the dynamically controllable thermal insulators that can be adaptable in compliance with environmental changes. For this reason, Choe et al. presented a smart hairy skin for adaptive thermal insulation and camouflage in the infrared (IR) range (Fig. 5b) [102]. Inspired by a hairy structure that serves to block the heat transfer from the epidermis to the surrounding by the air layer between hairs standing upright, the as-presented hierarchically porous shape memory polymers (SMPs) could control the degree of thermal insulation by standing or lying depending on the temperature condition due to its shape memory characteristics. Note that the modulus change of SMPs around glass transition temperature is a key factor in determining shape memory behavior. Moreover, a hierarchically porous structure can also switch between pore-open and pore-closed states by the modulus change. Benefitting from the synergic effect of hair posture (standing and lying states) and pore (open and closed states) modulation, the hairy SMP composites were capable of modulating the thermal insulating performances (dynamic modulation of thermal insulation around 61.4%); at the hair-standing/pore-open states, the SMP composites exhibited high thermal insulation, and on the other hand, the hair-lying/pore-closed states decreased the thermal insulating performance. These smart hairy SMP composites with tunable thermal insulation properties hold promise for future thermoregulation applications such as personal thermal management, industrial energy-saving technologies, and wearable IR camouflages [15].

Similarly, numerous studies have investigated the development of wearable thermal insulators for personal thermal management. However, the need for a high-temperature-resistant thermal insulator that can withstand harsh environments remains a significant challenge. To address this issue, recent research efforts have focused on enhancing the mechanical robustness of inorganic aerogels, known for their high-temperature resistance, while preserving their beneficial properties. One of the major challenges in developing inorganic aerogels as robust wearable thermal insulators is their intrinsic brittleness. Therefore, researchers have explored novel approaches to enhance the mechanical properties of inorganic aerogels to make them suitable for a wide range of high-temperature applications [16, 17, 103–106]. Thus, Dou et al. provided bendable and compressible ceramic nanofibrous aerogels by integrating flexible SiO_2_ nanofibers with interwoven celluloses (Fig. 5c) [18]. Notably, architectural continuity is a key factor in determining mechanical robustness. The assembly of the silica nanofiber with a high aspect ratio and 3D structured celluloses achieved improved structural continuity, thereby exhibiting superior buckling and compressive recovery (up to 85%). Benefiting from the robust property, the ceramic aerogels presented temperature-invariant superelasticity over a broad temperature range (-193 ~ 1,100 °C). Moreover, the aerogels exposed to the flame of the butane torch showed a slow temperature increase to 110 °C. They maintained an almost constant temperature in the later minutes, implying the potent possibility for high-temperature thermal insulator applications. However, achieving mechanical robustness alone is insufficient for ceramic-based thermal insulators to be practical for wearable thermal management. Further efforts to achieve stretchability are necessary because human skin can stretch up to 25% [19]. Therefore, researchers should focus on developing ceramic-based thermal insulators that not only possess mechanical robustness but also exhibit stretchability, making them suitable for wearable thermal management applications.

### Photothermal Effect

Light energy, such as sunlight, is a great source of heat. One absorbs light energy by exposing one’s skin or clothes to light. This absorption endows heating and thereby thermal regulation. However, much of the light energy is reflected or dissipated from one’s body without photothermal effect devices. To be specific, the light energy is divided into three modes when it hits a surface. It is absorbed, reflected, or transmitted. Since there is a limited amount of light energy, it is necessary to absorb as much light energy as possible to fully exploit the photothermal effect and achieve thermal regulation by warming one’s body. Therefore, in order to achieve thermal regulation by the light source, it is essential to come up with an effective photothermal effect device. The photothermal effect refers to the conversion of light energy into heat energy through a process of absorption and conversion. When a material is exposed to light, the photons are absorbed by the material, which then converts the light energy into thermal energy or heat. In recent decades, researchers have explored various materials for photothermal applications, including carbon-based materials, semiconductors, and plasmonic metals. Among them, carbon-based materials exhibit numerous conjugated *π* bonds, readily excited in the range of solar light, thereby absorbing the solar light in a wide range of wavelengths. In this regard, Peng et al. presented a densely arranged laser-induced graphene (LIG) film to enhance photothermal efficiency [107]. Inspired by the forest structure, hierarchical porous LIG films comprising micro- and nanostructures can trap and absorb the light efficiently, thereby minimizing the light reflection. However, despite the high efficiency of the solar-heating effect, the forest-like LIG film is unsuitable for wearable photothermal applications due to its poor mechanical stability, limited flexibility, and discomfort during wear. Thus, comfortable wearability must be addressed for solar-to-heat conversion technology for wearable applications.

**Fig. 6:**
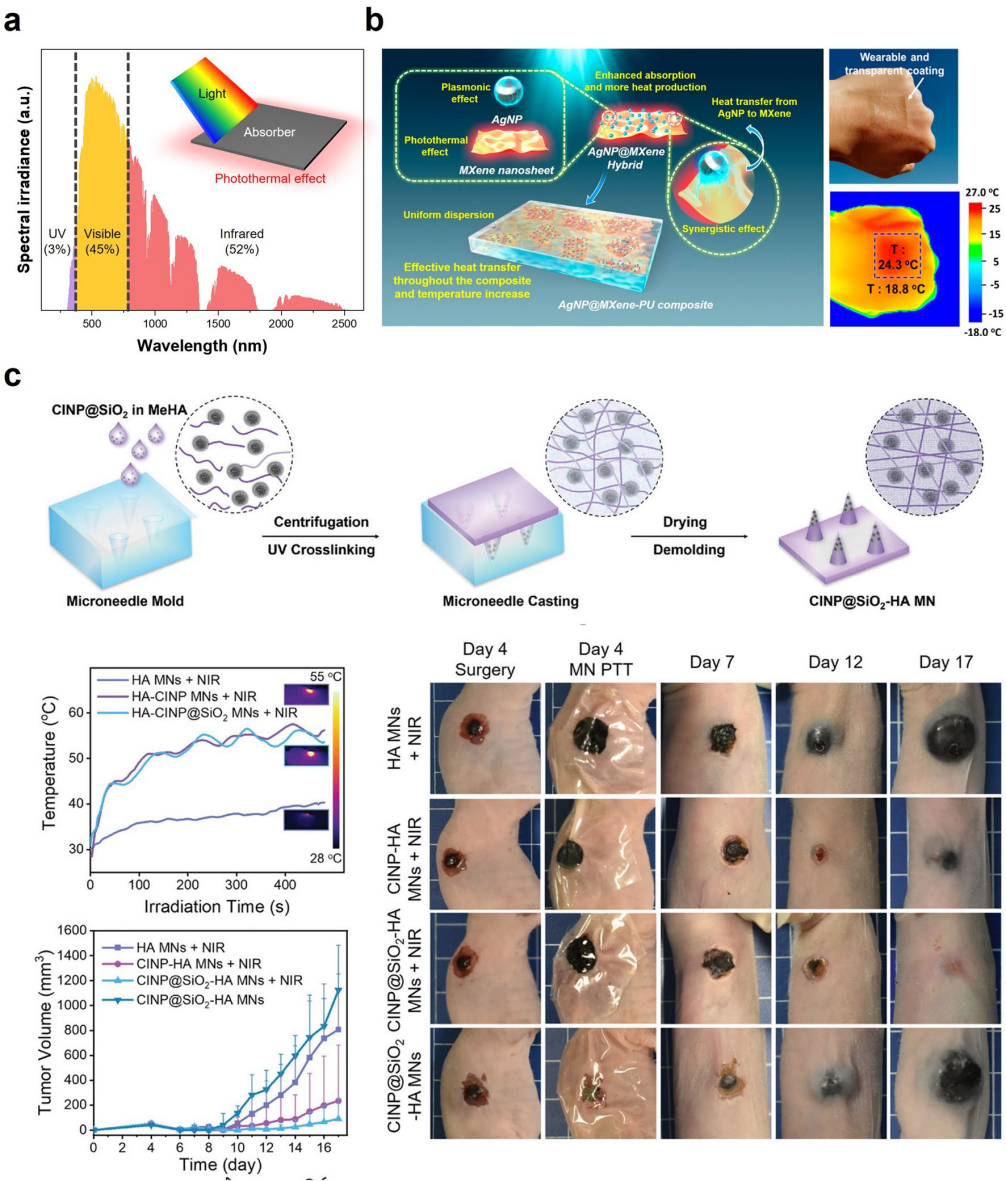
Photothermal effect-based passive thermal management: **a** AM 1.5 G solar spectrum. The inset shows the schematic illustration of heated material by photothermal effect. **b** Wearable and transparent MXene & AgNP coating with light-driven healable properties. Reproduced with permission [20]. Copyright 2019, ACS publications. **c** Microneedle patches using bio-based materials for photothermal therapy. Reproduced with permission [21]. Copyright 2022, Wiley–VCH

Meanwhile, state-of-the-art wearable devices often malfunction due to the device's vulnerability to external stimuli. In this regard, the healable materials-based wearable device has attracted significant interest in recent decades. The photothermal effect can be a promising trigger for healing procedures due to invasive repairs. Thus, Fan et al. provided a healable and transparent wearable device based on plasmonic silver nanoparticle (AgNP)@MXene nanosheet composites, as shown in Fig. 6a [20]. The wearable composite film showed excellent light-to-heat conversion efficiency due to the synergistic effects of plasmonic AgNPs and thermally conductive MXene. Under light irradiation for 5 min, the temperature of the film rose to approximately 111 ± 2.6 °C, making it a promising material for solar-to-heat conversion applications. Furthermore, the composite exhibited outstanding healing efficiency (> 97%), as the cracked AgNP@MXene-polymer composites could recover their original shape within a few minutes. The wearable healable composite coating can provide a powerful solution to the prolonged-usable wearable electronics due to the light-driven noncontact healing property (Fig. 6b).

Light-to-heat conversion technology has been widely employed as a promising therapeutic strategy for wound healing applications. In this regard, Lei et al. have developed microneedle patches functionalized with melanin nanoparticles for tumor photothermal therapy (PTT) and wound healing (Fig. 6c) [21]. Melanin nanoparticles (MNPs) derived from cuttlefish ink exhibit promising properties for photothermal therapy, including outstanding photothermal and antioxidative functionalities. To improve biocompatibility and promote skin generation, the MNPs were encapsulated in silica, which decomposes to release SiO_4_^4−^. The resulting MNPs@SiO_2_ demonstrated excellent photothermal characteristics, with a temperature increase of 60 °C at 100 µg mL^−1^ under near-infrared (NIR) laser irradiation (808 nm, 1 W cm^−2^). Benefitting the photothermal conversion property of MNPs@SiO_2_, the in vivo photothermal therapeutic effects were investigated in nude mice bearing tumor cells. The tumor cells in the mice with the microneedle patches decreased in size. The surgical wound was fully healed on day 17, implying the excellent PTT of the microneedle patches without burn damage on epidermis tissue. Thus, the photothermal effect-based therapeutic strategy will significantly contribute to a potent remedy for wound healing applications. This work broadened the scope of wearable thermal management, showing that thermal managing technologies can be utilized for therapeutic and medical purposes instead of simply maintaining thermal homeostasis.

Besides, tremendous efforts have focused on photothermal technology for wearable thermoelectric generators, thereby enhancing the energy-harvesting efficiency of thermoelectric generators [22]. For instance, Jeong et al. provided a wearable thermoelectric generator that consists of the top side as a transparent PDMS substrate and the bottom side as a solar-absorbing black substrate, improving the power-generating efficiency by the enhanced temperature difference due to the light-to-heat conversion ability of the bottom substrate [23]. Meanwhile, beyond a single heating functionality of the photothermal effect, a myriad of research has been investigated on wearable heaters based on the photothermal effect and joule-heating (detailed information about joule-heating is presented in the later section). For instance, Li et al. reported on developing MXene-based wearable heaters, which both solar and electric power can drive [18]. The hierarchical crest-ridge structure of MXene allows for efficient light absorption (up to 93.2%) and multiple internal reflections, resulting in a temperature increase of up to 65.4 °C in an equilibrium state and excellent photothermal performance. The high electrical conductivity of the MXene coating (2,000 S cm^−1^) enables it to function as a wearable heater, providing dual heating capabilities with both solar and electric inputs. On the other hand, Shi et al. combined the multifunction of heating such as joule heating, solar heating, and radiative heating for personal thermoregulation [25]. In summary, photothermal conversion technology has gained great interest in wearable thermal management, such as healable devices, healthcare management through PTT, thermoelectric generation, and dual heating sources.

### Sweat Evaporative Thermoregulation

The human body regulates its temperature through perspiration as part of homeostasis. However, if sweat is not effectively removed from the skin, it can accumulate and lead to thermal discomfort, compromising an individual's thermoregulation ability. Conventional hydrophilic textiles, such as cotton, can absorb sweat, but the moisture on the fabric can be critical for effective thermoregulation. In this regard, appropriate management of sweat evaporation is important for personal thermoregulation. To address this issue, numerous efforts have concentrated on directional water transport based on the Janus wettability structure comprising one side with hydrophilicity and the other with hydrophobicity. The wettability gradient by Janus architecture facilitates the sweat extraction from the inner hydrophobic to the outer hydrophilic side without sweat accumulation, thereby enabling the human body to maintain homeostasis.

**Fig. 7:**
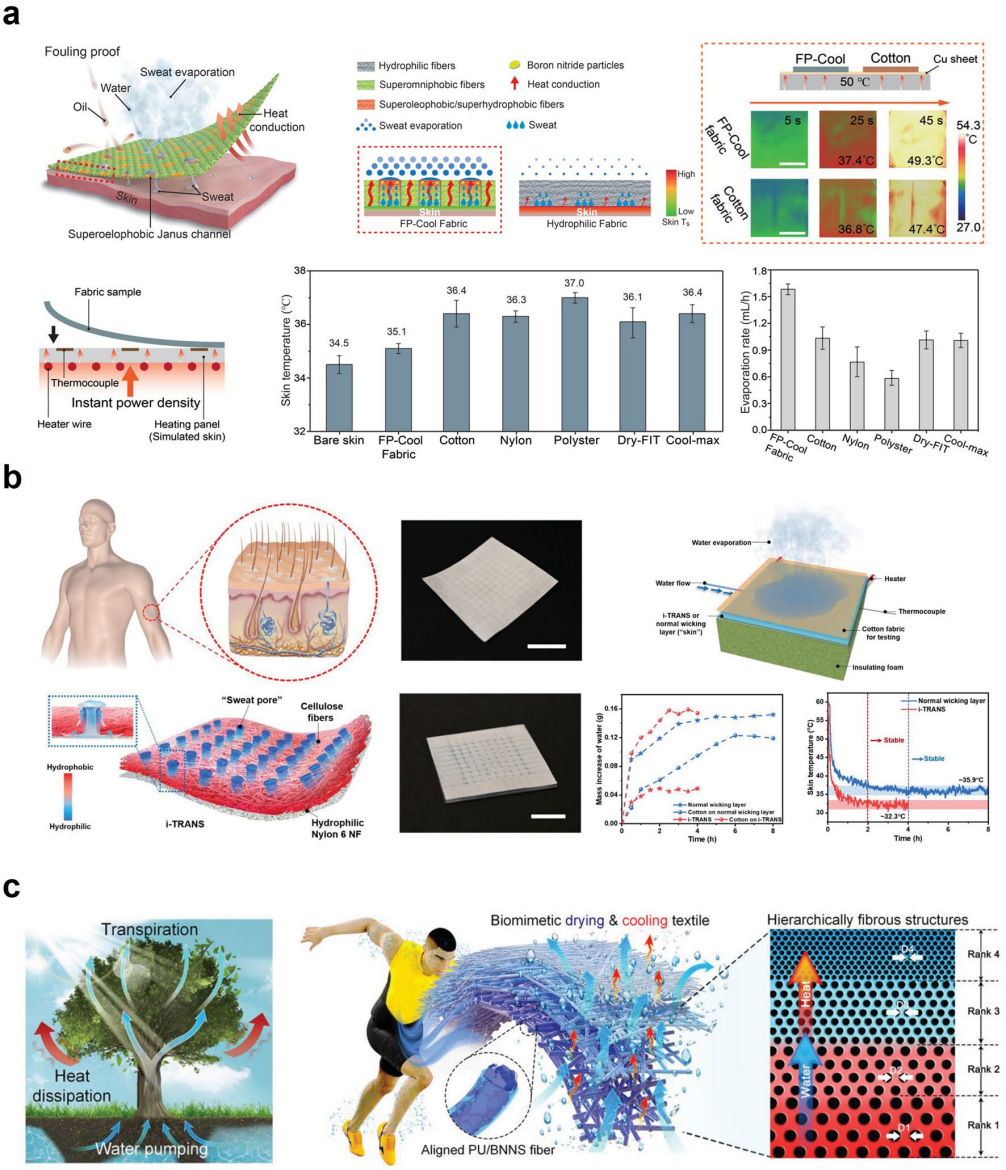
Sweat evaporation for passive thermoregulation: **a** Fouling-proof cooling (FP-Cool) fabric with sweat-wicking functionality for personal cooling. Reproduced with permission [26]. Copyright 2022, Wiley–VCH. **b** Hydrophobic/hydrophilic designed artificial sweating skin inspired by human body respiration. Reproduced with permission [27]. Copyright 2022, Wiley–VCH. **c** Biomimetic transpiration textile with one-way water transport. Reproduced with permission [29]. Copyright 2021, Wiley–VCH

Therefore, researchers have focused on developing textiles that exhibit wettability gradients to enhance both thermal and wet comfort. For instance, He et al. developed a skin-friendly Janus textile based on natural silks, showing two distinct surface properties—one side is hydrophilic, and the other is hydrophobic [108]. In this work, the naturally hydrophilic silk fabric was dip-coated in a hydrophobic solution followed by single-side plasma treatment to realize the Janus structure of hydrophobicity-hydrophilicity. Meanwhile, the duration time of the hydrophilic effect by plasma treatment relies on the type of textiles, exposure time to plasma, textile storage conditions, and type of plasma treatment. The hydrophilicity of the textile endowed by plasma treatment recovers to a lower surface energy state (hydrophobic state) after a few hours at least or weeks at most, depending on the external environment such as the condition of usage and storage [109]. The result demonstrated that the Janus silk fabric temperature on the skin was lower than that of pristine silk (~ 1.5 °C) during exercise. Moreover, Li et al. developed a cooling fabric with spatially-placed Janus channels for personal thermal management (Fig. 7a) [26]. The cotton fabric, which was treated with several chemicals such as poly(diallyldimethylammonium chloride) (PDDA), poly(sodium 4-styrenesulfonate) (PSS), and pentadecafluorooctanoic acid (PFOA), exhibited superomniphobic property. Additionally, to develop island Janus channels on the single-side of the fabric, the UV light was irradiated on the fabric, functionalizing the fabric with oxygen-containing hydrophilic groups. The fouling-proof cooling (FP-Cool) fabric featured the one-way water transport (positive direction), while preventing the negative directional transport. Benefitting the directional sweat flow, the FP-Cool fabric exhibited 50% higher evaporation rate than conventional textiles.

In addition to the functionalization of the single textile for Janus wettability, numerous approaches integrated several materials in recent years. Peng et al. developed an integrated hydrophilic/hydrophobic design with selectively distributed sweat pores (Fig. 7b) [27]. Inspired by human body perspiration, the diluted poly(dimethylsiloxane) (PDMS) was spatially coated on the hydrophilic membrane with a gradient in the vertical direction. The as-prepared design, which was optimized by controlling PDMS solution concentration, showed the stable temperature of artificial skin lower than that with a normal wicking layer (~ 3.6 °C).

Also, natural objects have inspired many researchers to develop the engineered design facilitating one-way sweat transportation. For instance, Dai et al. demonstrated cone-shaped micropores-structured Janus textiles inspired by the capillary phenomena of shorebird beaks [110]. The conical pores embedded in Janus textile were prepared by ablation of hydrophobic polyester (PE)/hydrophilic nitrocellulose (NC) membrane. The novel architecture of Janus textiles, in which asymmetric pores were engineered, exhibited an ultrahigh directional water transport capability (1,246%). Based on the biomimetic Janus wettability, the skin temperature covered with the PE/NC membrane showed an average temperature of 24.3 °C in a wet condition, which is higher than that achieved with traditional cotton textiles (21.7 °C). This phenomenon mainly originates from the low thermal conductivity of Janus textile compared to that of conventional cotton in wet conditions, as Janus textile is capable of being dry even in wet conditions due to its spontaneous one-way directional water transport functionality [110]. These results demonstrate the capability of Janus textiles to provide a warmer sensation to individuals and enhance their thermal comfort. Meanwhile, inspired by mussels that exhibit a remarkable capability of sticking to submerged rocks, Wang et al. developed the Janus-structured PET fabric based on cation-*π* interaction [28]. Several researchers have proven that cation-*π* interaction enhances the underwater adhesion of mussels [111], thereby extensively developing underwater sticky materials. The cation–*π* hydrophilic agent (CPHA) was readily stuck on the PET surface due to the active cation-*π* interaction by the rich aromatic moieties in the PET fibers, which renders the strong adhesion property, thereby converting the single-side of the PET surface from hydrophobic to hydrophilic. The mussel-inspired fabric possessed a remarkable directional water transport ability (1,115%) based on Janus wettability, demonstrating a more excellent cooling effect than conventional cotton and original PET fabrics. In addition, Miao et al. proposed a hierarchical fibrous structured textile for liquid transportation by mimicking vascular plants (Fig. 7c) [29]. The hierarchically structured membrane with capillary pores decreasing in size achieved a large capillary force without resistance to water transport. Moreover, the aligned polyurethane (PU)/boron nitride nanosheet (BNNS) fibers, which contact the human epidermis, served as the thermally conductive path. Benefitting from the favorable properties for drying and cooling, the multilayered textiles exhibited excellent directional water transport capability (1,072%), rapid liquid evaporation rate (0.36 g h^−1^), and high thermal conductivity (out-of-plane ~ 0.182 W m^−1^ K^−1^, in-plane ~ 1.137 W m^−1^ K^−1^). Likewise, the technology modulating sweat evaporation combined with enhancement of thermal conductivity is a promising approach for efficient personal thermal management. In this regard, numerous researchers have explored the efficient approach to one-way water transportation based on Janus wettability.

Lastly, a recent study demonstrated the integrated cooling (i-Cool) textile that combined a thermally conductive path and water transport channels to enhance the evaporation rate and cooling effect [30]. The heat-conducting path efficiently transported thermal energy from the body to the evaporation spots, further accelerating the sweat evaporation into the air due to the synergistic effect of the main functionalities (conductive heat path and water channel). Compared to conventional textiles, i-Cool textiles exhibited lower skin temperature and a more rapid evaporation rate. Hence, many studies concentrated on efficient personal perspiration management based on various approaches, providing significant insights for future perspiration-based thermo-regulative textiles.

### Radiative Cooling

Radiative cooling (RC) technology is a promising candidate for passive thermal management due to its passive property that consumes no additional energy input to dissipate the heat [112]. In recent decades, the RC method, which emits heat to the cold outer space through the atmospheric window (λ ~ 8 to 13 μm, where λ is the wavelength), has been extensively developed for passive daytime radiative cooling (PDRC) by simultaneously reflecting solar irradiance. Yet, several PDRC designs based on photonic structures [113], polymers [114], and metamaterials [115] reduce the potent possibility of personal thermal management due to poor comfortable wearability. In this regard, an extensive range of research has been devoted to employing the PDRC techniques to be appropriately applied for personal cooling management by addressing the issue of wearable comfort [116].

**Fig. 8:**
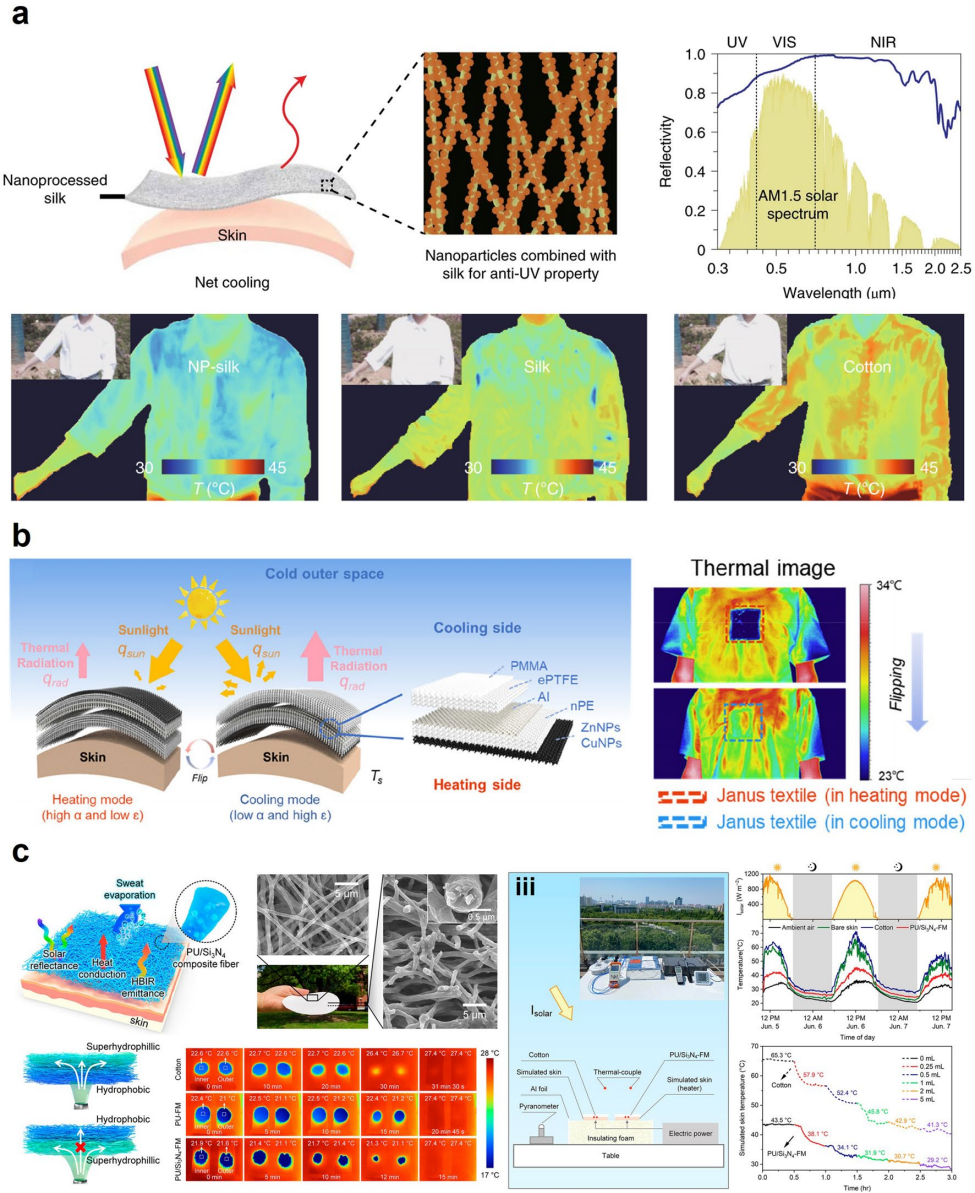
Personal radiative cooling for passive thermal management: **a** Nanoprocessed silk-based radiative cooling textile. Reproduced under the terms of CC BY [31]. Copyright 2021, Springer Nature. **b** Janus textile with radiative cooling and solar heating functionalities for all-day outdoor personal thermal management. Reproduced with permission [33]. Copyright 2021, ACS publications. **c** Hierarchical fibrous membrane that utilizes radiative and evaporative heat dissipation for enhancing the cooling performance. Reproduced with permission [37]. Copyright 2022, ACS publications

Zhu et al. provided silk-based PDRC textiles with comfortable wearability, as shown in Fig. 8a [31]. The silk, which exhibits a cooling and comfortable sensation on the human epidermis based on a natural-based hierarchical structure, has been regarded as a suitable material for wearable RC application due to the high reflectivity in the visible (VIS) to near-infrared (NIR) range and high emissivity in the mid-infrared (MIR) spectrum and yet, a critical limitation still remains challenging for PDRC application; high absorption in the ultraviolet (UV) region. To address this issue, silk was dip-coated using tetrabutyl titanate (TT) as a coupling reagent and aluminum oxide (Al_2_O_3_) that exhibits a minimum absorption in the UV range. As a result, the reflectivity of silk was enhanced from 70 to 85% in the UV range, and that of silk in the VIS–NIR region was also improved from 86 to 95%. Benefitting from the enhanced reflectivity in the UV–VIS-NIR region and high intrinsic emissivity in the MIR range, the nanoprocessed silk exhibited a temperature drop of ~ 3.5 °C in the daytime. Moreover, the simulated skin temperature covered with the nanoprocessed silk textile was ~ 8 and ~ 12.5 °C lower than the skin covered with natural silk and cotton, respectively. Functionalizing the natural material will open a novel energy-saving personal thermal management strategy with comforting wearability.

Moreover, Zeng et al. presented a large-scale hierarchical structured metafabric for scalable PDRC that cooled a human body ~ 4.8 °C lower than one covered with commercial cotton fabric [32]. Likewise, numerous researchers have employed PDRC technology for wearable personal thermal management. In addition to the personal thermoregulation application, the PDRC technology has been dedicated to enhancing the various electricity-generating modules such as solar-cell [117] and thermoelectric generators [118]. Among them, wearable thermoelectric generators (TEGs) have extensively employed the PDRC structure to maximize the thermal gradient of TEG and enhance the energy-harvesting efficiency, thereby sustainably powering wearable electronics [22, 119, 120]. In this regard, Khan et al. introduced the integration of poly(vinylidene fluoride-co-hexafluoropropylene) (P(VdF-HFP)) radiative-cooled films to a wearable TEG [121]. The porous P(VdF-HFP) films, fabricated via the phase separation method, exhibited excellent solar reflectivity (96.7%) and high emissivity (97.47%) within the IR range. The films effectively reduced the daytime temperature by 6 °C below the ambient temperature. Benefitting the favorable radiative cooling property, the TEG was integrated into the porous film, which served as an efficient heat sink with comfortable wearability due to the micro-thin thickness (~ 800 μm), achieved the enhanced power generation (~ 12.48 μW cm^−2^) with the daytime temperature difference of 1.37 °C higher than that of TEG with a commercial fin-type heat sink (~ 1.25 °C).

Furthermore, for the PDRC technology to be practically applied, several studies have focused on functionalizing the radiative coolers with solar-heating properties, which can switch between dual modes depending on the environmental condition [122, 123]. For instance, Luo et al. demonstrated a wearable bi-functional Janus textile that is capable of facilitating dynamic passive personal thermoregulation of solar-heating and radiative-cooling (Fig. 8b) [33]. The Janus textile rendered one side with a heating function and the other side with a cooling function; the heating side consists of copper (Cu) and zinc (Zn) nanoparticles (NPs), which exhibits high solar absorption (> 80%) due to the plasmonic resonances and low MIR emittance (~ 16%). The cooling side is comprised of porous polymethylmethacrylate (PMMA) covered expanded polytetrafluoroethylene (ePTFE) on the Al layer, which enhances the solar reflection (~ 91%) and the MIR emissivity (87%). Profiting from the optical properties, the Janus textile in heating mode exhibited a simulated skin temperature of 8.1 °C higher than the skin simulator covered with conventional cotton. In cooling mode, the temperature decreased by 6 °C compared to conventional cotton.

On the other hand, to improve the passive cooling performance beyond a single function of RC, the combined technology of RC with the other passive cooling method has been extensively developed in recent decades. Among them, the synergy of SE and RC has emerged as promising candidates for passive cooling management [34–36]. For instance, Miao et al. demonstrated a hierarchically designed membrane based on polyurethane/silicon nitride (PU/Si_3_N_4_) fiber with Janus wettability (Fig. 8c) [37]. The fibrous membrane exhibited high solar reflectance (91%) and IR emittance (93%) due to the high refractive index of Si_3_N_4_ and intrinsic bond vibrations of polymer/inorganic membranes, respectively. Moreover, a single side of the membrane achieved hydrophilicity by plasma treatment, enabling directional water transport from the hydrophobic to the hydrophilic side, facilitating sweat evaporation, and improving the evaporative cooling effect. Benefiting from these characteristics, the PU/Si_3_N_4_ fiber-based membrane showed a substantial temperature drop of 21.9 °C compared to conventional cotton.

## Active Thermal Management

### Joule-heating-based Thermal Management

Joule-heating, or resistive heating, elevates a temperature of an electrical conductor by kinetic energy transfer by electrons. When an electron is affected by a certain electric field, it gains kinetic energy. The collision of the electron with particles in the conductor induces a kinetic energy transfer and, eventually, a certain amount of heat energy. Since the total kinetic energy of an electron is proportional to the current and the voltage, the heating power is proportional to the product of the two [124]:

4 $$P\propto VI=\frac{{V}^{2}}{R}$$

where *V*,* I* and *R* are electrical voltage, current and resistance respectively. Even though bidirectional thermal management, or heating and cooling, by a single joule heating device is not possible, it is still an attractive thermal management unit for many wearable devices due to a simple heating mechanism and a facile application by simply connecting an electrical conductor with the power source. Also, obtaining high temperatures by putting more electrical energy into the heating conductor is not difficult [125–129].

**Fig. 9:**
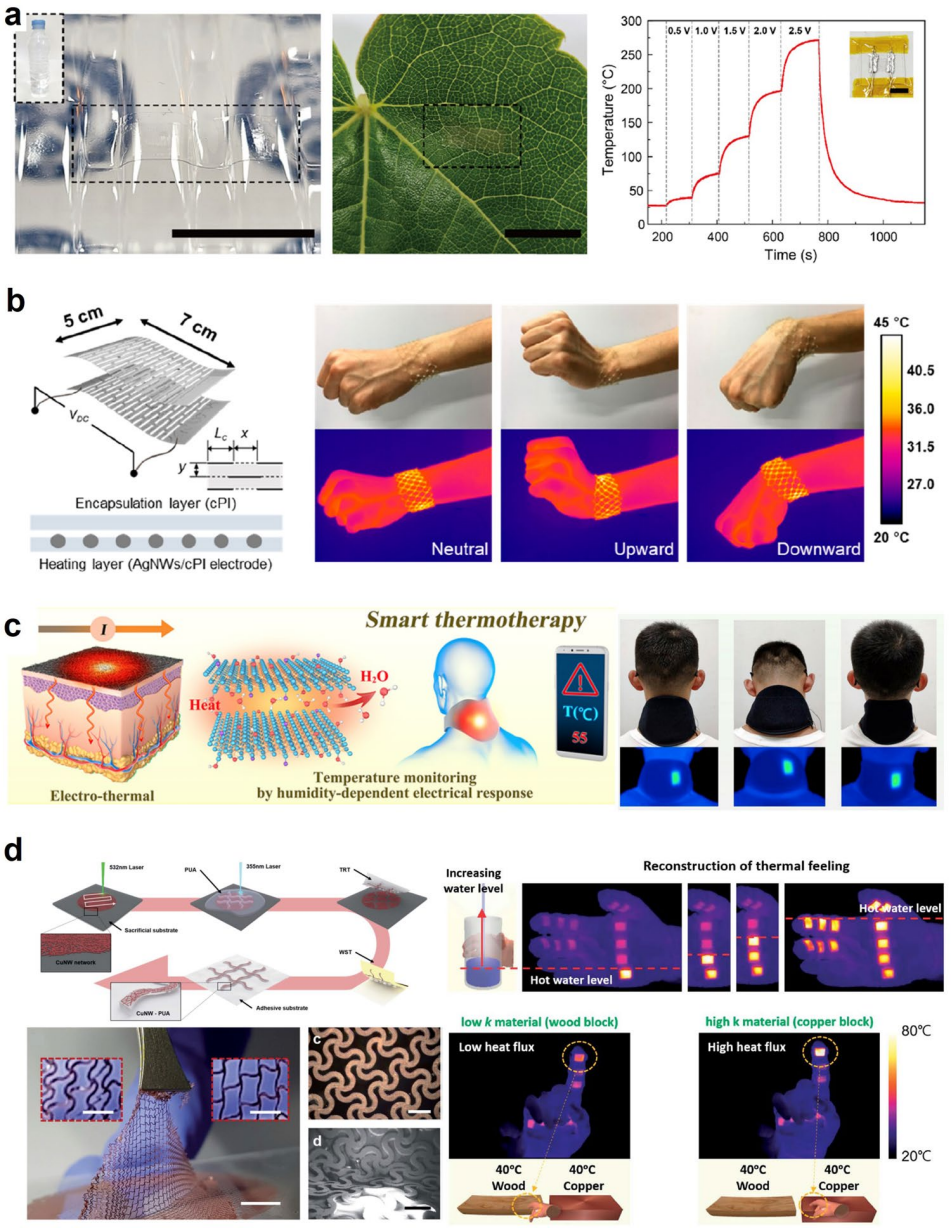
Joule heating for active thermal management: **a** Transparent liquid metal electrodes for the heater. Reproduced with permission [38]. Copyright 2022, ELSEVIER. **b** Stretchable and transparent Kirigami patterned electrodes for the heater. Reproduced with permission [39]. Copyright 2019, ACS publications. **c** Smart MXene fabric heater for healthcare and medical therapy. Reproduced with permission [40]. Copyright 2020, ACS publications. **d** Highly stretchable Cu nanowire heater for virtual reality applications. Reproduced with permission [41]. Copyright 2020, Royal Society of Chemistry

Owing to a simple component of a joule-heating-based device, an electrical conductor, researchers have been fabricating transparent heaters with the mechanism [130–134]. The transparency endows the heater with many abilities, such as watching the affected area while practicing thermotherapy, defrosting for windows, and applying with imperceptibility. Additionally, they have granted the heaters with softness, stretchability, and transparency to realize wearable transparent thermal management units. In doing so, they had to develop methods for minimal temperature change while being applied to a soft surface with mechanical deformation. This could be achieved by minimal resistance change under deformation since the heating power, which is proportional to the temperature of the heater, is directly related to the resistance under the same voltage. For example, Yun et al. presented transferable transparent liquid metal electrodes with grid structure for heaters. Manufacturing an opaque material to have a grid structure with extremely narrow linewidth is one of the typical ways to endow transparency to a device. They used a direct printing method to fabricate the liquid metal grid with a linewidth lower than 5 μm. The transmittance was as high as 90.1% due to the small linewidth. The as-fabricated heater obtained a temperature of 272 °C with an input voltage of 2.5 V due to its low sheet resistance. The heater worked without any failure under a maximum mechanical strain of 100%. It even was able to be operated in an − 30 °C environment, maintaining 89 °C with an input voltage of 1.4 V. The researchers explained that the proposed heater with low sheet resistance and high stretchability is expected to be utilized in thermal management film for windows (Fig. 9a) [38].

Won et al. demonstrated another transparent heater research operated by a joule-heating mechanism. This time, they fabricated the heater with a silver nanowire percolation network since it possesses intrinsic transparency. Moreover, they introduced Kirigami-patterning, which cuts the thin film with the desired pattern to endow extreme stretchability. In this way, they achieved a transparent and stretchable heater. They manufactured a wrist band type heater that can perform stably under various mechanical deformations. Since the Kirigami heater had electromechanical decoupling, it could work stably under 200% strain without any operational degradation. The heater could reach over 80 °C with an input voltage of 9.0 V. The researchers expect this heater to be utilized for wearable thermal haptic, wound healing monitoring, and personal thermal treatment with transparency and stretchability (Fig. 9b) [39].

Zhao et al. demonstrated a smart multifunctional fabric with fast humidity response and joule heating for medical therapy as an example of a personal thermal treatment application. The fabric possessed a smart system that could be utilized as bacterial ablation for healing an infectious wound while having an alarm system for a low-temperature burn. They fabricated the fabric by coating MXene on a nonwoven cellulose fiber. The fabric had breathability with excellent mechanical stability. Firstly, the group devised a fabric-integrated neck-guarding pad. The pad functioned well when positioned on one’s neck. Furthermore, they exploited the humidity-temperature-resistance relation to manufacturing a smart and safe heater system. They developed a built-in alarm to turn on an LED when the heater's temperature exceeds a certain degree. They also verified that the heating induces bacterial ablation and accelerates wound healing performance (Fig. 9c) [40]. This research showed that Joule heating contributes to thermoregulatory wearables. Such a mechanism can be employed to expand the applications of heaters in various fields, including the medical area.

Due to the simple, repetitive, and precisely controllable feature of joule-heating, it can be utilized in fields of virtual reality (VR) as a thermo-haptic device because it can readily control the localized temperature of the user. Hence, it should be considered one of the scopes of thermal management applications. For instance, Kim et al. replicated a feeling of heat in a VR space with a copper nanowire (CuNW)-based heater. To utilize the heater for VR applications, they encapsulated easily oxidized CuNW with polyurethane-acrylate (PUA) to increase the stability and oxidation-resistivity. The as-fabricated heater was patterned into the serpentine structure that granted a high stretchability with small resistance change, which was beneficial for the thermo-haptic device that required precise temperature control. It exhibited a stable repetitive operation with a mean temperature of 120 °C under an input voltage of 7 V. It also demonstrated stability under the stretching condition without apparent temperature variance. They fabricated a thermo-haptic glove by mounting 12 heaters on a nylon glove. They calculated the heat flux that transfers to one’s skin when touching a 40 °C copper and a 40 °C wood. Then, they computed the required temperature for the heater to transfer the same amount of flux to the skin. Finally, they replicated the heat sensing while touching different materials in the VR world, verified by IR image. In addition to that, they also replicated the sense of heat for approaching a fire and holding a cup with increasing water level. The researchers emphasized that the simple mechanism of joule-heating with accurate temperature control with only changing input voltage could realize this highly precise thermo-haptic device for VR space (Fig. 9d) [41]. Although the study mentioned above focused on the VR application, the identical material and manufacturing technologies can be directly applied to make a digitalized heater that provides thermoregulatory functionality.

Kim et al. developed a biomimetic chameleon soft robot realized by a heater and thermochromic liquid crystal as another application for the joule-heating-based heater. The research group developed a vertically-stacked, multi-layered heater component that mimics chameleon skin's crypsis and disruptive coloration. Each heating layer was patterned differently to obtain diverse coloration patterns by controlling each layer. The group used an AgNW percolation network for each layer on a colorless polyimide thin film. Since the film was extremely thin, the heater in each layer had negligible performance differences. With the low sheet resistance of the heater film, the temperature could reach 43 °C with an input voltage of 0.7 V. The heater exhibited improved stability by implementing a proportional-integral-derivative (PID) control-based feedback system to control the input power. It could maintain its target temperature even when the surrounding temperature decreased due to the presence of nearby ice. The group successfully demonstrated the camouflage of a chameleon-inspired soft robot with manufactured skin. Additionally, they installed a color sensor at the bottom of the robot to sense the color of its standing floor and change the skin color accordingly. As a proof of concept, the robot was placed on a floor with red, green, and blue colors and successfully changed color while walking through the different color regions [42]. Even though its main application was devoted to robotics, this work demonstrated that the PID-control integrated heater technology offers insights into thermal managing wearables because the device can transfer a controlled amount of heat to the user regardless of the external temperature.

### Micro-fluidic based Thermal Management

**Fig. 10:**
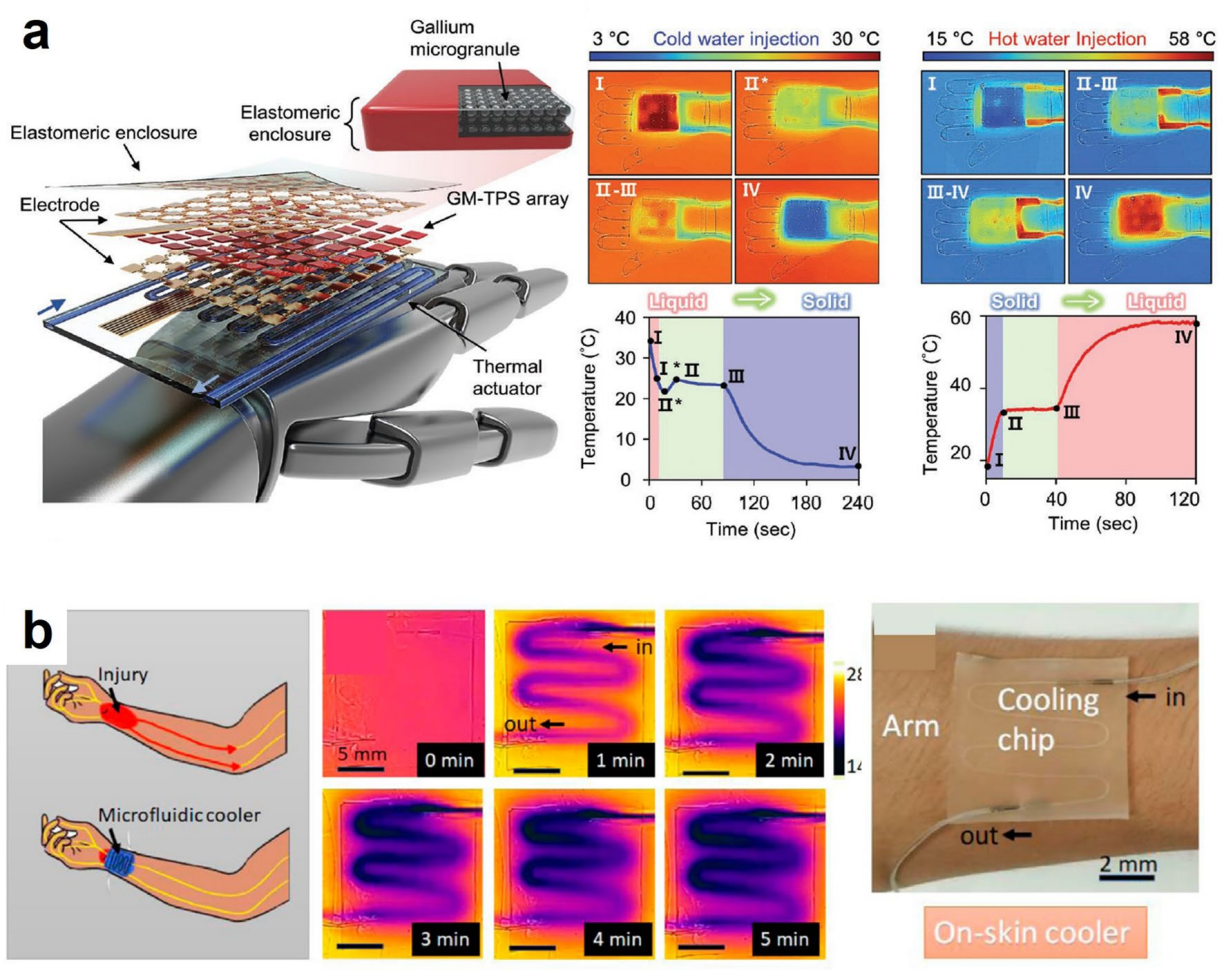
Microfluidic cooling for active thermal management: **a** An adaptive robotic skin with a microfluidic cooling device. Reproduced with permission [43]. Copyright 2022, Wiley–VCH. **b** A liquid metal mold-based 3D flexible microfluidics [45]. Copyright 2022, Frontiers Media

Micro-fluidic thermal management is a method to cool or heat a target surface by exploiting the conduction and convection of fluid in a micro-channel [61, 135–139]. To achieve thermal management, a microfluidic channel must be attached to a target surface. Then, a refrigerant or heating fluid is injected and flows through the channel while heat flux between the target surface and the fluid occurs. Researchers in this field further utilize phase change of the fluid to maximize its cooling or heating performance, similar to a heat pump. Also, they can achieve bi-directional thermal management by simply replacing the fluid with different temperatures. The simple bi-directional thermal managing mechanism and its microscale device with flexibility facilitate wearable applications for one’s nerve or skin. As a representative example, Lee et al. applied micro-fluidic thermal management to control adaptive robotic skin with a sensor. The group fabricated a capacitive sensor that consists of two electrodes and a dielectric layer sandwiched between the two. The dielectric layer was composed of an elastomer with gallium microgranules embedded inside. The gallium microgranule, which had a melting temperature of 29.76 °C, was utilized as a sensing performance shifting agent by phase-change between liquid and solid. When the microgranule was in the solid state, the capacitive sensor could possess a high detecting range of pressure. On the other hand, the sensor could possess high sensitivity when the microgranule was in a liquid state. The group fabricated an array of these sensors to be applied to a robot hand with two distinct sensing abilities. And a thermal actuator, or a thermal management device, employed in hand was a microfluidic channel with water. The facile alteration of target temperature by simply changing the water temperature in the channel was why they chose this management device. Thereby, they could readily manipulate the phase of the gallium microgranule by substituting water with different temperatures. They injected cold water at 1 °C for solidification of the granule and hot water at 65 °C for liquefication of it. It took less than a minute to induce the phase transition achieving an adaptive robotic skin with two distinct capacitive sensing abilities (Fig. 10a) [43].

With its small scale and facile application, the micro-fluidic channel could realize pain management by thermally managing the peripheral nerve system. Reeder et al. devised soft, bioresorbable microfluidic coolers that could be directly implanted into a nerve and eliminate the neural activity to manage one’s pain. They fabricated the microfluidic device with all bioresorbable components so that it could be readily implanted and utilized without the necessity of removal. The device consists of soft evaporative microfluidic channels for nerve cooling and a temperature sensor for real-time temperature monitoring. They chose a liquid coolant of perfluoro-pentane (PFP) with a superheating ability with a boiling temperature of 30 °C. The coolant underwent its phase transition when mixed with N_2_ gas in a serpentine evaporative channel placed on a target cooling surface. The device achieved a minimum nerve temperature of − 1.4 °C. The maximum cooling rate was 3 °C s^−1^ with its optimized mixing ratio and flow rate. Animal experiments proved the cooling device could eliminate nerve signals realizing on-demand analgesia [44]. Similarly, Yan et al. developed a wearable thermal management system using 3D flexible microfluidics. The group utilized gallium to fabricate microfluidic channels that were directly applied to the wrist such that cold water at 0 °C could be circulated through the channel to cool the skin surface. With a flow rate of 1.75 mL min^−1^, the average temperature of the skin surface was reduced from 35.3 to 20.3 °C. The authors suggest this device could be useful for medical applications, such as post-surgery recovery or injury treatment. (Fig. 10b) [45].

### Caloric Material

Caloric effect-based thermal management, which utilizes a caloric material that undergoes a great change in entropy under magnetic, electric, or mechanical fields (uniaxial or isotropic), is rising as a promising candidate for substituting conventional refrigeration systems [140–144]. The conventional system possesses many limitations considering current environmental issues such as [143] global warming, abnormal climate, la Niña phenomenon, and the goal of carbon neutral society. It must utilize harmful refrigerants to achieve a cooling effect by phase change. Not only do refrigerants harm the environment, the mechanism of conventional refrigeration inevitably demands a massive size of a refrigerator since it has to undergo a phase transition inside the device. In this situation, the caloric effect cooling device must substitute the conventional device.

Caloric effects refer to reversible thermal changes in materials that respond to magnetic, electrical, or mechanical stimuli when exposed to changes in applied magnetics, electric, or mechanical fields (uniaxial or isotropic). These thermal changes are quantified as adiabatic temperature change Δ*T*, isothermal entropy change ΔS, and isothermal heat *Q*. Conventional caloric materials show Δ*T* > 0, Δ*S* < 0, and Q < 0, and inverse caloric materials show Δ*T* < 0, Δ*S* > 0, and *Q* > 0, respectively when the magnitude of the driving field is increased. Caloric effects are classified as magnetocaloric (MC), electrocaloric (EC), and mechanocaloric (mC), which are driven by changes in the magnetic field (*H*), electric field (*E*), and stress field (*σ*), respectively. In principle, any material can generate a reversible temperature change when acted upon by an adiabatic stimulus. However, ferroic materials exhibit spontaneous electric or magnetic ordering of the caloric effects and are characterized by large and reversible entropy changes at phase transitions. In traditional ferromagnetic (FM) or ferroelectric (FE) materials, large MC or EC effects are observed near their Curie temperatures above which the materials become paramagnetic or paraelectric with no respective long-range magnetic or dipolar ordering. During such reversible phase transitions, the ferroic material undergoes a transition from an ordered (low entropy) state to a disordered (high entropy) state with large thermal change. Given these attractive intrinsic properties, various studies have been conducted on thermal management systems utilizing such MC and EC materials.

#### Magnetocaloric-based Thermal Management

The MC cooling device utilizes a magneto-thermodynamic phenomenon that takes advantage of an entropy change under a magnetic field. The expression for the phenomenon is as follows [145]:

5 $$\Delta {T}_{\mathrm{ad}}=-{\int }_{{H}_{0}}^{{H}_{1}}{\left(\frac{T}{C\left(T,H\right)}\right)}_{H}{\left(\frac{\partial M\left(T,H\right)}{\partial T}\right)}_{H}\mathrm{d}H$$

where, $\Delta {T}_{\mathrm{ad}}$ is the adiabatic temperature change, *T* is the system's temperature, *H* is the applied magnetic fields, *C* is the heat capacity of a refrigerant, and *M* is the magnetization of the refrigerant.

A qualitative explanation for the phenomenon is as follows. When an external magnetic field is applied to the magnetocaloric material, its magnetic dipoles are aligned to a certain orientation. This causes the entropy of the material to decrease, thereby inducing an increase in temperature by the adiabatic temperature change to maintain the total entropy. On the contrary, when the field is removed from the magnetocaloric material, the entropy of the material increases with decreasing temperature. This phenomenon can be readily utilized in a cooling system if a coolant diminishes the increased temperature under a magnetic field. Once diminished, removing the applied magnetic field will induce the material to cool down further than before the magnetic field was applied. This phenomenon can be utilized to fabricate a cooling device with the MC effect.

**Fig. 11:**
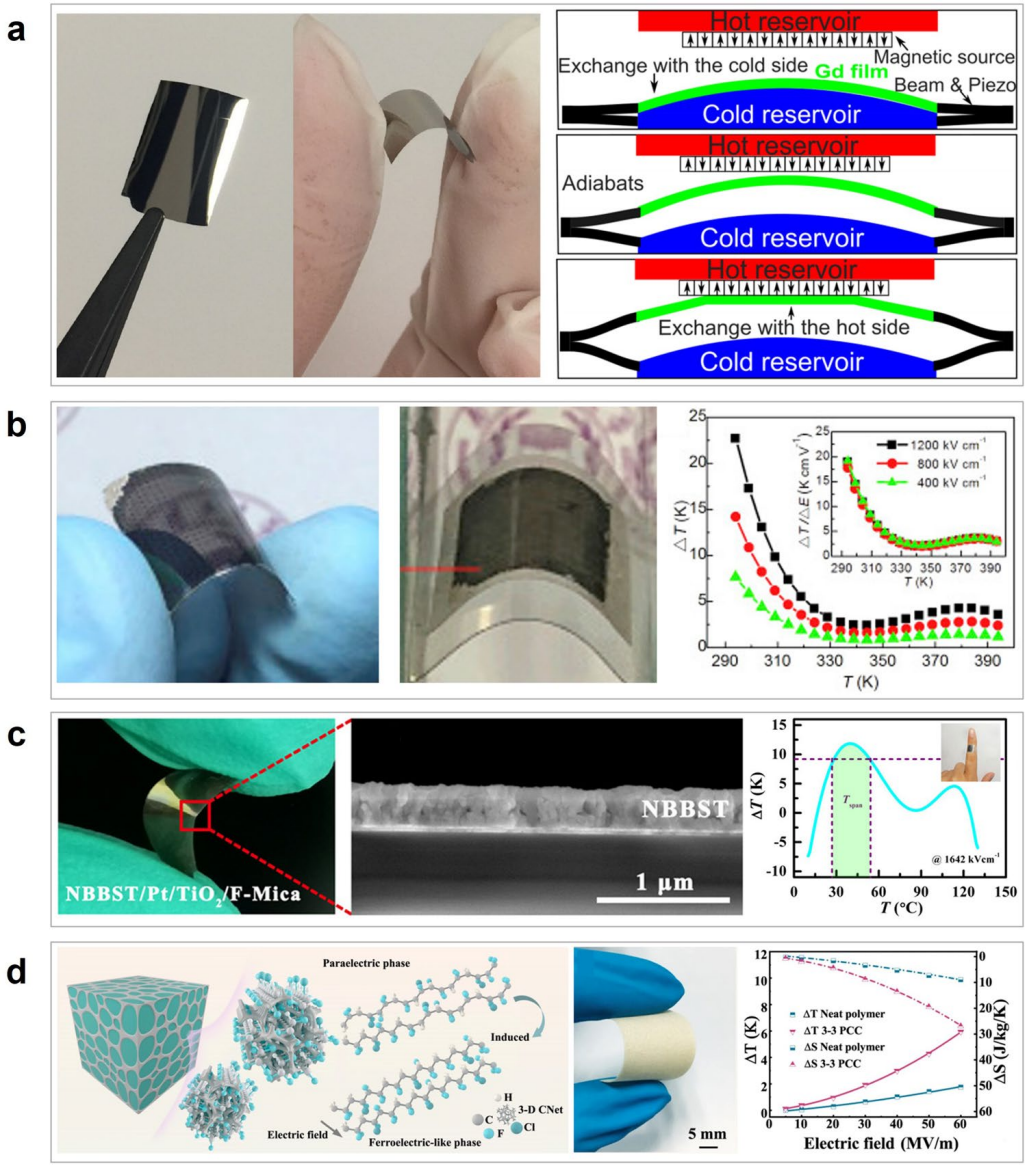
Caloric effect for active thermal management. **a** Flexible and freestanding gadolinium film for magnetocaloric applications [46]. Copyright 2020, Wiley–VCH. **b** Pb0.82Ba0.08La0.1Zr0.9Ti0.1O3 (PBLZT) thin film‐based flexible EC device [104]. Copyright 2019, Elsevier. **c** 0.65(0.94Na0.5Bi0.5TiO_3_-0.06BaTiO_3_)-0.35SrTiO_3_ (NBBST) film‐based flexible EC device [49]. Copyright 2020, ACS Publications. **d** Ba0.85Ca0.15Zr0.1Ti0.9O_3_ (BCZT) ceramic network—P(VDF-TrFE-CFE) polymer matrix composite EC device. Reproduced with permission [107]. Copyright 2022, Springer Nature

Since the range of working temperature depends greatly on the Curie temperature of MC materials, we will focus on near-room-temperature working MC materials for this review article [146–150]. Gd, or Gadolinium, is the representative material for a room-temperature MC material because it has a high magnetocaloric effect of 7.6 ${\upmu }_{\mathrm{B}}$ per atom near room temperature (since Curie temperature of Gd is 294 K). Due to its near-room-temperature applications, researchers in this field are eagerly developing practical Gd-based MC thermal managing devices for medical implants and wearable devices [151–154]. For instance, Ba et al. fabricated a free-standing polycrystalline Gd flexible film with a smooth surface that can bend reversibly with no MC properties degradation. The maximum entropy change was measured as 10.5 J kg^−1^ K^−1^ at H_f_ of 7 T, similar to the Gd bulk. The group claims that conventional heat exchange fluid and pumps for MC cooling devices become unnecessary using this thin film structure, allowing direct solid–solid contact for heat exchange. They expect the thin MC material film will pave a new way for microscale, high-efficiency devices such as the Internet of Things and medical implants when combined with an appropriate actuation system such as a piezoelectric beam (Fig. 11a) [46].

Current MC researchers mainly focus on finding appropriate alloy or ceramic materials with higher MC properties for substituting conventional refrigerating systems with less attention to wearable devices. Nevertheless, it is quite expected that the advent of a new wearable thermal management system utilizing the MC effect will soon arrive, too. Recently, researchers in this field are endeavoring to develop a flexible MC device, an appropriate power system for the magnetic field, an actuator with minimal power consumption, and a miniaturized complete device such as the one introduced above. The research is expected to eventually be utilized to develop a new MC-based wearable thermal managing device in the near future [155–157].

#### Electrocaloric-based Thermal Management

The EC effect shares many of the same underlying mechanisms as the MC effect. When an electric field is applied to an EC material starting from ${E}_{0}$=0, its constant entropy causes its temperature to increase. The material's entropy decreases as the heat generated by the electric field is transferred to a heat sink in a constant field. When the electric field returns to zero, the material undergoes adiabatic depolarization, causing its entropy to increase again and resulting in a cooling effect. This process involves repeated cycles of heat transfer between the target material and the heat sink. The Maxwell relation, combined with the pyroelectric effect, can be used to create a generic equation for the EC effect, which describes the adiabatic change in temperature from an initial value of ${E}_{0}$ to a final value of ${E}_{1}$. The adiabatic change in temperature from an initial value of ${E}_{0}$ to a final value of ${E}_{1}$ can be described as follows [158]:

6 $$\Delta {T}_{\mathrm{ad}}=-{\int }_{{E}_{0}}^{{E}_{1}}\frac{T}{{C}_{E}}\left(\frac{\partial P}{\partial T}\right)\mathrm{d}E$$

where $D={\varepsilon }_{0}E+ P$ where *E*, *P*, and ${\varepsilon }_{0}$ correspond to the electric field, polarization, and vacuum dielectric permittivity (${\varepsilon }_{0}$ = 8.85 × 10^−12^ F m^−1^), respectively.

Researchers have investigated a wide range of inorganic and organic materials for their potential to be used in efficient cooling devices. Inorganic EC materials are typically perovskites, with many different types of perovskites being studied, including PbTiO_3_ (PT)-based, BaTiO_3_ (BT)-based, KNbO_3_ (KN)-based, SrTiO_3_ (ST)-based single crystals and ceramics [159, 160], solid solutions, and several 2D inorganic materials. This large pool of inorganic ferroelectric materials has made them attractive candidates for use in EC cooling devices. However, these inorganic materials have disadvantages such as high cost, low processability, and low mechanical elasticity [161].

Polymeric dielectric films, including poly(vinylidene fluoride) (PVDF)-based materials, have been studied for use in EC cooling due to their low cost, high electrical breakdown strength, mechanical elasticity, and high processability [162]. PVDF-based EC materials, such as semi-crystalline poly(vinylidene-trifluoroethylene) (P(VDF-TrFE)) (50/50 mol%), have gained attention due to their strong polarization originating from the spontaneous orientation of dipoles in the crystalline phase [163]. Although PVDF-based EC materials have a lower Curie temperature than conventional ceramic films, their Curie temperature is still higher than the human body temperature, making them suitable for wearable thermal management applications. Moreover, compared to inorganic materials, polymeric EC materials have the disadvantage of requiring higher voltage. Therefore, large EC effects under low electric fields are critical for practical applications such as wearable coolers and on-chip thermal management. The high voltage needs to be avoided, and the external electric field is set to approximately 25–40% of the breakdown field [161].

Manipulating defects in the material system is a strategy for reducing the Curie temperature and required voltage in EC materials. This can involve modifying the defect structure or converting normal ferroelectric P(VDF-TrFE) into P(VDF-TrFE-CFE) or P(VDF-TrFE-CFE-CTFE) tetrapolymers, which enhances the degree of freedom and broadens the original F-P transition temperature, shifting it closer to room temperature [159]. For example, at 29.9 °C, the P(VDF-TrFE-CFE) (59.2/33.6/7.2 mol%) terpolymer showed a Δ*T* of 7.6 and 14.8 °C under 100 and 150 MV m^−1^, respectively [164]. Similarly, at 34 °C, the P(VDF-TrFE-CFE-CTFE) (56.1/33.2/5.2/4.8 mol%) tetrapolymer exhibited a Δ*T* of 8 °C under 100 MV m^−1^ [165].

Another effective way to enhance the ECE is by introducing a built-in field, which amplifies the ECE response for the same ∆E when the initial field E0 is higher [161]. Such built-in fields can be induced by incorporating nano-fillers such as organic and inorganic FE nano-regions [166] or nanoparticles [167, 168], and 2D materials with well-aligned structures [169]. The interfacial charges that arise at the matrix-filler boundaries also contribute to the dipolar manipulation of the material. These strategies offer a promising path toward developing more efficient and effective EC materials suitable for various practical applications, such as on-chip thermal management and wearable cooling devices.

To successfully develop wearable thermal management systems, in addition to effective and strong cooling power at low temperatures and electric fields, a flexible device structure is required to improve heat transfer efficiency without restricting the user's movement. One promising approach for such devices is using lead-free ceramic materials that offer high performance with enhanced mechanical properties compared to rigid ceramic thin-film structures. For example, vertically aligned Ba_0.67_Sr_0.33_TiO_3_ (BST) nanowire arrays have been employed to produce a strong cooling power of 15 W g^−1^ at low temperatures, with lead-free ceramic nanostructures exhibiting Δ*T* values of 10.1 and 3 °C in a voltage range safe for human use [47]. Researchers have also investigated using Pb_0.82_Ba_0.08_La_0.1_Zr_0.9_Ti_0.1_O_3_ (PBLZT) inorganic thin films, demonstrating high performance with a ΔT of 22.5 K at room temperature (Fig. 11b) [48]. Cyclic fatigue tests on the PBLZT thin film suggested its potential as an EC material for wearable thermal management systems.

Another example is an Mn-modified 0.65(0.94Na_0.5_Bi_0.5_TiO_3_-0.06BaTiO_3_)-0.35SrTiO_3_ (NBBST) film, which did not mechanically break down even after 104 bending cycles with a 5 mm tensile/compressive bending radius (Fig. 11c) [49]. The NBBST film had a maximum Δ*T* of 12 K at 40 °C and a Δ*T* of 9 °C in a low electric field, operating over wide temperature ranges without significant performance degradation. These results demonstrate the potential of lead-free ceramic materials for use in wearable EC cooling devices with high cooling power, good mechanical properties, and a wide range of operating temperatures. A composite structure is also attractive for fabricating flexible EC devices. For example, Pure perovskite structured Pb_0.8_Ba_0.2_ZrO_3_ nanofibers (PBZ-nfs) obtained by electrospinning were compounded with PVDF to fabricate composite film through the solution casting method [50]. The fiber structure not only makes the device flexible but also increases interface polarization, thereby enhancing the electrocaloric effect. The EC composite film exhibited a 1.62 times larger EC effect than pristine PVDF. The ΔT and ΔS of the composite film are 13.99 K and 52.70 J kg^−1^ K, respectively, at 150 MV m^−1^ and − 30 °C. Then when the temperature rose to 70 °C, ΔT and ΔS (entropy) became 5.08 K and 13.56 J kg^−1^ K, respectively. Recently, Li et al. introduced Ba_0.85_Ca_0.15_Zr_0.1_Ti_0.9_O_3_ (BCZT) ceramic network into P(VDF-TrFE-CFE) polymer matrix (Fig. 11d) [51]. The porous ceramic network has good flexibility and increases the number of polar nanodomains. It also enhances interfacial areas of the polar/nonpolar phases and ceramic network/polymer, increasing manipulable entropy at low fields. On the other hand, the continuous 3-D network structure opens up phonons' high-speed thermal conduction path at the “hot spots” formed by the nucleation of nanodomains, which enables rapid cold/heat transport in the electrocaloric layer. The resulting material exhibits a 240% increase in electrocaloric performance and a 300% enhancement in thermal conductivity compared to the neat polymer. While the author applied this EC device to the cooling of 5G chips, the flexibility and high cooling power of this material demonstrate its attractiveness as a material for wearable EC thermal management devices.

Wearable EC cooling devices with high EC stability and wide operating temperature ranges have been developed. Still, heat transfer between the EC material and the heat sink and between the material and the skin is crucial for efficient use in wearable thermal management systems. Circulating fluids like air or water can be used for heat exchange, but it is challenging to implement this in wearable THDs because of the need for a bulky mechanical pump [47, 170]. Ma et al. proposed an alternative approach [171]. They used the interesting electrostatic actuation system of the flexible EC material to periodically initiate and break thermal contact with the heat source and heat sink, creating net heat pumping. This method has been realized using a flexible P(VDF-TrFE-CFE) film, which showed a specific cooling power of 2.8 W g^−1^ and COP of 13, with a flexible device structure that could autonomously transfer heat between the heat source and heat sink. The EC systems mentioned earlier are innovative, but they have a drawback. They would require a relatively large heat sink to dissipate heat over a prolonged period to generate a cooling effect, limiting the device's wearability. Despite significant progress in device efficiency, operating temperature, and flexible device structure, the miniaturization of the heat sink remains a significant challenge for the successful implementation of EC systems in wearable thermal management devices.

### Thermoelectric-based Thermal Management

The thermoelectric effect comprises three distinct physical mechanisms: the Seebeck effect, the Peltier effect, and the Thomson effect. The Seebeck effect arises when two dissimilar conductors form two separate contact points at different temperatures, resulting in an electromotive force due to the shift in electron energy levels. On the other hand, the Peltier effect is the converse phenomenon of the Seebeck effect. When an electrical current flows in the same configuration as the two conductors but has no temperature difference, heat is generated at one point and absorbed at the other. Lastly, the Thomson effect occurs when there is a temperature gradient within a single conductor, leading to heating or cooling, depending on the material's properties [172–174].

Since our main focus of the review article is wearable thermal management, we will spotlight the Peltier effect, commonly used to actively achieve both heating and cooling by simply changing the direction of the input current. Bismuth telluride-based alloys are most commonly utilized when fabricating a practical thermoelectric device [175–182]. since they have the highest thermoelectric figure of merit, or zT, due to their high electrical conductivity, low thermal conductivity, and a huge Seebeck coefficient at room temperature. Also, it is innocuous, relatively cheap, and applicable for ambient room temperature conditions. Therefore, researchers have utilized pellet-type bismuth telluride-based alloys for their practical applications. Since the pellets are rigid and have a cuboid shape, the fabrication of soft wearable devices with these pellets and methods to enhance their thermoelectric working efficiency have become the most important issue in the research area. Simple equations expressing the heating and cooling in a thermoelectric device are as follows [162]:

7 $${\mathrm{Q}}_{\mathrm{c}}=2N\left(\alpha I{T}_{\mathrm{c}}-k\mathrm{\Delta T}-\frac{1}{2}{R}_{\mathrm{e}}{I}^{2}\right)$$

8 $${\mathrm{Q}}_{h}=2N\left(\alpha I{T}_{\mathrm{h}}-k\mathrm{\Delta T}+\frac{1}{2}{R}_{\mathrm{e}}{I}^{2}\right)$$

where ${Q}_{\mathrm{c}}$, and ${Q}_{\mathrm{h}}$ refer to the heat generated in cooling and heating mode. $\mathrm{N}, \alpha , I, {T}_{\mathrm{c}}, {T}_{\mathrm{h}},\Delta T, {R}_{\mathrm{e}}$ and $k$ refer to the number of pellets, the Seebeck coefficient, current, cold side temperature, hot side temperature, temperature difference between the two, electrical resistance, and thermal conductivity, respectively. Shortly, the basic thermoelectric heat generation is represented by the first term in the equation. The second and third terms represent heat transfer between two sides and the Joule heating effect, respectively. There exist many ways to enhance the efficiency of the thermoelectric device. Firstly, researchers find various ways to minimize the heat transfer from the hot to the cold side. The device is often encapsulated to grasp the tiny pellets to the electrical conductors stably. However, this encapsulating material, commonly an elastomer, fills the surface of pellets and enhances heat conduction from the hot to the cold side.

To minimize this conduction, researchers have devised some techniques, such as encapsulating with porous elastomer or no encapsulating at all with another method to achieve stability of the device. Secondly, researchers can implement a heat sink on the hot side to prevent heat conduction to the other side and heat accumulation such that the sink facilitates the heat to flow in the designated direction so that unwanted parasitic heat loss does not occur. The heat sinks frequently utilized are based on a fin-type thermal conductor, phase transition materials, or highly conductive elastomers. Thirdly, researchers aim to achieve high stretchability to ensure stable operation under mechanical deformations and to enable highly conformal contact. For practical wearable thermoelectric devices, it is crucial to maintain intimate contact with the irregular surface of human skin. This contact reinforces heat conduction between the skin and the device, ultimately maximizing the device's efficiency. To achieve these features, soft and stretchable conductors, as well as heat sinks, are utilized. Lastly, researchers focus on developing methods to maximize the fill factor of thermoelectric pellets to attain a high areal density of the device. Since the number of pellets directly affects the amount of heat, achieving a high fill factor is crucial. To accomplish this, researchers have developed a highly efficient structure to lay as many pellets as possible and an automatic machine that picks and places the pellets in a manner that cannot be achieved manually, thus maximizing the fill factor.

**Fig. 12:**
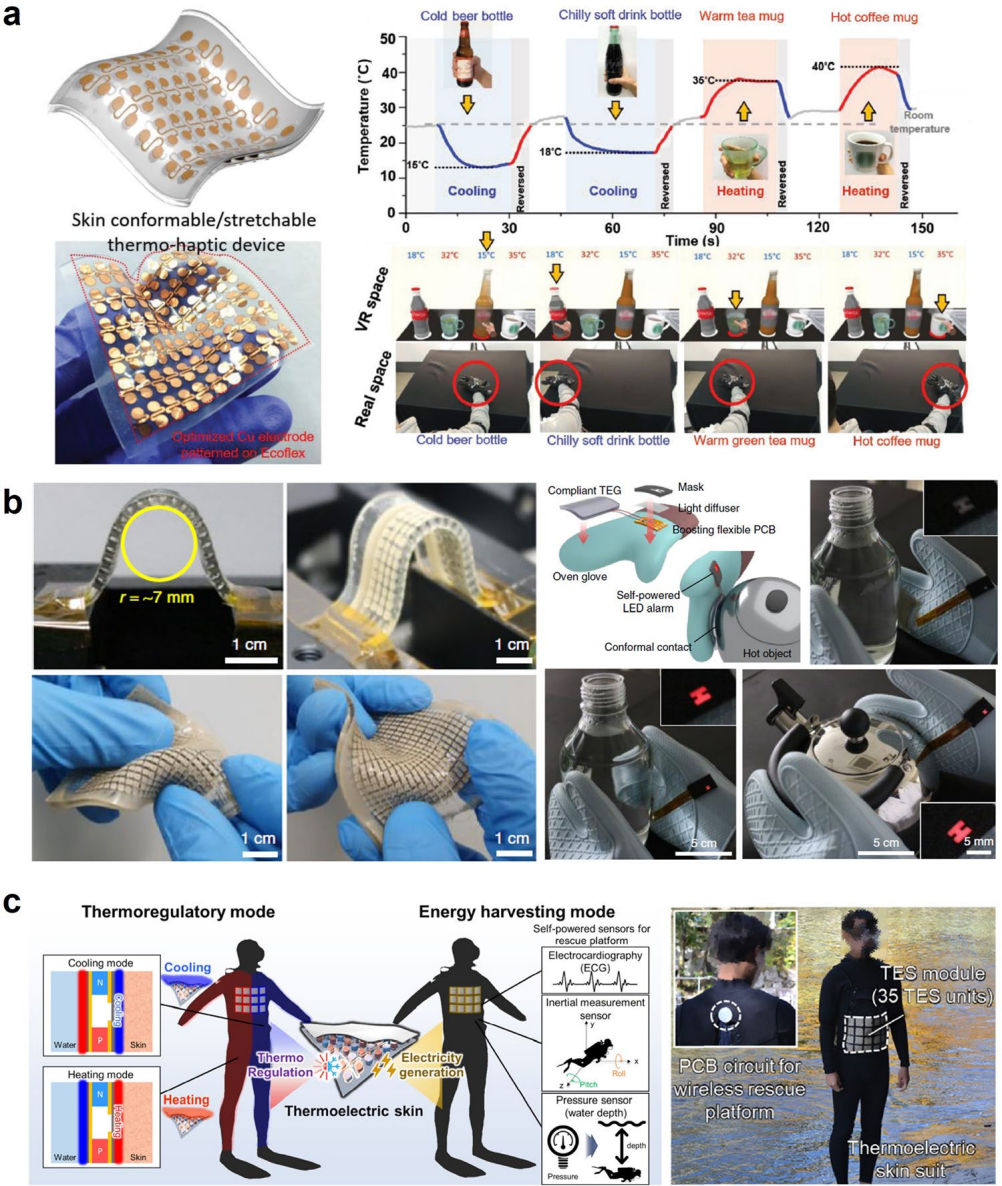
Thermoelectric effect for active thermal management: **a** Stretchable skin-like thermoelectric device for virtual reality applications. Reproduced with permission [52]. Copyright 2020, Wiley–VCH. **b** Soft and compliant thermoelectric generators with high performance. Reproduced with permission [54]. Copyright 2020, Springer Nature. **c** Soft multi-modal thermoelectric skin [55]. Copyright 2022, Elsevier

For example, Lee et al. developed a thermoelectric-based glove to create an artificial heat sensation in virtual reality. They successfully reconstructed the sensation by carefully modulating the direction and amount of current flowing through the device. As a proof of concept, they demonstrated that the sense of heat transferred from the device could discriminate between holding a cold bottle and a hot mug. They also mimicked the heat sensation of holding paper, glass, and aluminum cups by considering the thermal conductivity of each material. They first patterned an optimized serpentine-shaped Cu electrode on an elastomer to fabricate the device and then soldered thermoelectric pellets to the conductor. Particularly, they tightly soldered both sides of the pellets to the conductor without encapsulation and utilized a thermally conductive elastomer on both sides to minimize heat loss. Compared to other control samples, the device exhibited significantly less parasitic heat resulting in higher efficiency for both heating and cooling [52] (Fig. 12a). Similarly, the group conducted another research using thermoelectric devices, fabricating an active imperceptible artificial skin. The skin was undetectable in the visible range during the day and in the infrared range at night. For the visible range, they utilized thermochromic liquid crystal that changed color based on the surrounding temperature and a thermoelectric device to regulate the temperature. They matched the device temperature with the background temperature for the infrared range, making it indistinguishable in infrared images. Although this work is not explicitly related to wearable thermal management, one of the distinguishable highlights in this work arises from the “pixelation” of the device such that each pixel can be independently controlled to transfer programmable heat. This work suggests that a thermoelectric device can be employed for localized cooling/heating of the human skin rather than thermally regulating the entire skin surface [53].

The careful fabrication of thermoelectric devices can lead to other uses utilizing the same device structure since the Seebeck effect is the reverse of the Peltier effect. For instance, Lee et al. fabricated a soft, compliant thermoelectric device with various techniques for self-powered wearable electronics. The group embedded the device in an alarm system glove and adopted various features to maximize thermoelectric efficiency. They fabricated a pellet pick-and-place machine to achieve a higher fill factor, resulting in a high areal density of pellets. They also utilized soft conductors for interconnection, which endowed the device with conformability and reduced the air gap between the target surface and the device. The presence of the air gap generally generates heat loss by accumulating heat. Moreover, they manufactured a vertically aligned Ag–Ni particle/PDMS elastomer to enhance heat conduction to the outer surface. Although they encapsulated the pellets with elastomer to obtain stability, the other factors mentioned above allowed the device to have high efficiency. (Fig. 12b) [54]. Jung et al. presented a soft thermoelectric skin with the functionalities of harvesting energy and regulating body temperature underwater as an example of utilizing both effects in one device. The research article describes the development of a soft, multi-modal thermoelectric skin that can harvest energy underwater while regulating body temperature. The device was designed using a composite of thermoelectric and hydrophobic materials that can function as both an energy harvester and a thermoregulator. The device demonstrated high flexibility, durability, and conformability, which make it suitable for wearable applications. The device was tested in a simulated underwater environment and showed promising results regarding its energy-harvesting capabilities and ability to regulate body temperature. The researchers believe this device has potential applications in underwater environments, such as for divers and aquatic animals, as well as other wearable applications requiring energy harvesting and thermoregulation. (Fig. 12c) [55]. To summarize, thermoelectric devices are an ideal candidate for human thermoregulation. They can generate active cooling and heating with a single device structure and transfer a large amount of heat to regulate the human body temperature.

However, the heat management of thermoelectric devices presents a common challenge that significantly impacts their energy conversion efficiency. The accumulation of excess heat in the presence of a significant temperature gradient across the device causes a decrease in performance, commonly referred to as the ‘heatsink problem’. An effective approach to mitigating the heatsink problem is utilizing a heat dissipation mechanism, such as a heatsink. However, the conventional use of metallic heatsinks presents inherent limitations, such as rigidity and bulkiness, which restricts their applicability in scenarios necessitating flexibility and conformability, such as wearable devices. To address the issue of heat dissipation, several research studies have investigated the use of flexible and stretchable materials such as thermally conductive fabric [183], phase-change materials [126, 184, 185], and hydrogels [13, 186, 187] to create heatsinks that can conform to the irregular surfaces of the thermoelectric devices while maintaining their thermal conductivity properties. Some of these studies have also explored the use of microfluidic channels embedded within the flexible heatsink to enhance the heat dissipation efficiency of the thermoelectric device [188]. Overall, developing flexible and stretchable heatsinks for thermoelectric devices is an active area of research that aims to address the heat dissipation challenges associated with these devices in various applications.

## Conclusion and Future Perspective

### Potential Barriers to be Widely Adopted in Commercial Markets

Wearable thermal managing devices are rigorously being developed due to the urgent need for the green solutions of approaching environmental issues. As discussed throughout this review paper, there are diverse methods to achieve wearable thermal management, and every method is quite effective. However, there are still some challenges to overcome for the devices to be widely adopted in commercial products. First, the total weight of the device should be as light as possible. For active devices, it is necessary to have an external source to realize thermal regulation. Therefore, it is important to minimize the external source gadget for lightening the weight. Though passive devices are lighter than active devices due to the absence of external sourcing gadgets, the device itself might be heavy to acquire enough effective thermal regulation. For instance, a material exploiting heat capacity or latent heat has better performance when it is heavier. It is important to develop materials with better intrinsic thermal properties. Second, it is essential for wearable thermal devices to have price competitiveness to penetrate the commercial market. This includes rational prices, long device lifespans, and massive productions. It is necessary to establish a fabrication process that can fulfill the aforementioned features. Moreover, since battery life or durability critically affects device lifespan, the development of highly efficient energy devices is important. Finally, a high degree of wearability must be satisfied to be prevalent in our society. The high degree of wearability means that it is as imperceptible and unbothered to the user as possible. Researches inventing devices that secure breathability, light-weight, and stretchability can be presented as examples for the high degree of wearability.

### Future Research Directions of Wearable Thermal Management Systems

Although many researches about wearable thermal management applications have been conducted, there are two future goals that this research field has to accomplish. One is to investigate new types of functional materials. The other is to integrate the existing wearable thermal management techniques with other wearable devices, such as sensory systems, energy harvesting systems or other types of thermal management devices. Discovery, or invention, of new materials for wearable thermal management, can impact various aspects. It allows the managing devices to be more efficient, comfortable, and effective. Moreover, various materials that have different working conditions can be chosen according to the operating environments. On the other hand, combining diverse wearable techniques together is necessary to fabricate practical and commercial devices. When thermal regulation systems are combined with sensory systems, they can effectively sense the status of the regulating target, and perform precise and required operations. Likewise, incorporating energy harvesting devices with thermal management systems can provide a self-supplying power source for thermal regulation. This will greatly reduce the bulkiness of thermal management systems that require a rather bothersome external power source. Lastly, integrating passive and active thermal management systems into one device can offer continuous and adaptable regulation. It can greatly broaden the device’s applicability according to one’s circumstances by turning on and off the active regulating device while the passive device is always working on its own.

### Conclusion

In this review, we methodically outline recent developments in thermal management wearable materials and cutting-edge methods for controlling body temperature. This research further classifies thermoregulatory wearables into various functional materials and technologies and splits them into two primary groups, namely active and passive thermal management. The strengths and limitations of each material/device that constitute each strategy are comprehensively discussed, followed by the future perspective and challenges of thermal regulatory wearable technologies. It is difficult to argue which thermal management mode should be preferred for wearable applications because each mode has clear strengths and weaknesses. For instance, even though active thermal management can directly regulate the body temperature and some active thermoregulatory technologies can provide active cooling and heating with a single device architecture, the active mode needs an external battery and the circuitry to provide the power source and control the device. On the other hand, while the passive mode does not require electricity, it cannot fine-tune the thermoregulatory performance. Thus, we believe that the careful choice of strategies and materials is needed for each situation because there are several options for human thermoregulatory purposes, as discussed. In addition, future material and technology advancements will help improve these devices' efficiency, making them more practical for long-term use. For instance, there is an invigorating interest in developing the deformable heatsink since one of the drawbacks of the thermoelectric device originates from its incapability to dissipate the accumulated heat. Moreover, for practical thermoregulation of the whole human body, large-area fabrication (i.e., a form of clothing) of devices can offer a solution so that these devices can cover the human body, thereby efficiently regulating the temperature of the entire body. On the other hand, the devices can thermally protect the localized area of the human body from external heat sources or cool down the specified spot of skin. In addition, we believe that a further combination of passive and active devices will generate synergetic thermoregulation functionalities as an efficient and novel thermal managing system. Also, we anticipate that integration with other technologies can offer many innovative opportunities for the future perspective of wearable thermal management applications. One potential direction is using machine learning and artificial intelligence to optimize real-time thermal management. This could lead to personalized temperature control based on individual preferences, activities, and physiological responses. Thus, in this regard, we expect that wearable thermal management technologies will advance further, providing users with improved comfort, performance, and health benefits.
